# The dependence of shugoshin on Bub1-kinase activity is dispensable for the maintenance of spindle assembly checkpoint response in *Cryptococcus neoformans*

**DOI:** 10.1371/journal.pgen.1011552

**Published:** 2025-01-13

**Authors:** Satya Dev Polisetty, Krishna Bhat, Kuladeep Das, Ivan Clark, Kevin G. Hardwick, Kaustuv Sanyal

**Affiliations:** 1 Molecular Mycology Laboratory, Molecular Biology and Genetics Unit, Jawaharlal Nehru Centre for Advanced Scientific Research, Bengaluru, India; 2 Institute of Cell Biology, University of Edinburgh, Edinburgh, United Kingdom; 3 Department of Biological Sciences, Bose Institute, Kolkata, India; Duke University, UNITED STATES OF AMERICA

## Abstract

During chromosome segregation, the spindle assembly checkpoint (SAC) detects errors in kinetochore-microtubule attachments. Timely activation and maintenance of the SAC until defects are corrected is essential for genome stability. Here, we show that shugoshin (Sgo1), a conserved tension-sensing protein, ensures the maintenance of SAC signals in response to unattached kinetochores during mitosis in a basidiomycete budding yeast *Cryptococcus neoformans*. Sgo1 maintains optimum levels of Aurora B kinase ^Ipl1^ and protein phosphatase 1 (PP1) at kinetochores. The absence of Sgo1 results in the loss of Aurora B^Ipl1^ with a concomitant increase in PP1 levels at kinetochores. This leads to a premature reduction in the kinetochore-bound Bub1 levels and early termination of the SAC signals. Intriguingly, the kinase function of Bub1 is dispensable for shugoshin’s subcellular localization. Sgo1 is predominantly localized to spindle pole bodies (SPBs) and along the mitotic spindle with a minor pool at kinetochores. In the absence of proper kinetochore-microtubule attachments, Sgo1 reinforces the Aurora B kinase^Ipl1^-PP1 phosphatase balance, which is critical for prolonged maintenance of the SAC response.

## Author summary

Precise segregation of the genome to daughter cells is the key to cell growth, survival, and proliferation. A proper attachment of spindle microtubules to the kinetochore of each eukaryotic chromosome is required for the faithful segregation of the genome. Error correction machinery such as the spindle assembly checkpoint (SAC) prevents chromosome mis-segregation by monitoring and correcting improper attachments by delaying the cell cycle progression. In this study, we probed the role of an evolutionarily conserved protein, shugoshin, in the basidiomycete budding yeast *Cryptococcus neoformans* in monitoring the kinetochore-MT attachments during mitosis. We show atypical localization of shugoshin to spindle pole bodies (SPBs) and along spindle MTs in this organism. Shugoshin, in *C*. *neoformans*, plays a role in maintaining the SAC response to prolong the mitotic arrest in MT depolymerizing conditions. Most strikingly, unlike other systems, the kinase activity of Bub1 is dispensable for the shugoshin’s localization and function during mitosis in *C*. *neoformans*. Overall, this work reveals the mitotic role of shugoshin in monitoring kinetochore-MT attachments in one of the most critically important human fungal pathogens.

## Introduction

The precise segregation of duplicated chromosomes in mitosis depends on the stable attachment of kinetochores with microtubules (MTs) and proper orientation on the mitotic spindle. The kinetochore is a multi-subunit protein complex composed of inner and outer layers [1]. The inner kinetochore assembles onto centromeric chromatin and is constitutively associated with DNA. Subsequently, the outer kinetochore assembles and promotes the attachment of MTs emanating from microtubule organizing centers: centrosomes in animals or spindle pole bodies (SPBs) in yeast species. Apart from playing a role in MT attachment, the kinetochore also acts as a signaling platform for recruiting mitotic surveillance components that detect unattached or improperly attached kinetochores [2–4]. Once errors are detected, cells remain arrested at metaphase until rectified.

Mitotic surveillance systems monitoring erroneous kinetochore-MT attachments include the spindle assembly checkpoint (SAC) and a chromosome passenger complex (CPC) component, Aurora B^Ipl1^. The SAC comprises Mad1, Mad2, BubR1, Bub3, and the kinases Bub1 and Mps1 [2]. The SAC machinery conveys a ’wait anaphase’ signal (MCC, the mitotic checkpoint complex) during cell division in response to unattached kinetochores that inhibits the anaphase-promoting complex (APC/C^Cdc20^) from ubiquitylating B-type cyclins and securin [5–7]. Upon the formation of proper kinetochore-MT attachments, the SAC is silenced. APC/C^Cdc20^ inhibition is relieved, resulting in the degradation of B-type cyclins and securin, thus promoting mitotic exit and sister chromatid separation [8,9].

Tension across sister-kinetochores attached to MTs is important in ensuring high fidelity of chromosome segregation. Tensionless kinetochore-MT attachments, if not rectified, result in the missegregation of chromosomes. Aurora B^Ipl1^ acts as a tension sensor, detecting tension across sister kinetochores [10–13]. In response to tensionless kinetochore-MT attachments, phosphorylation of outer kinetochore by Aurora B^Ipl1^ results in the destabilization of kinetochore-MT interactions, leading to the generation of unattached kinetochores [14,15] indirectly activating the SAC. Several reports implicate that Aurora B^Ipl1^ can also directly activate the SAC machinery in response to unattached kinetochores, indicating that the function of Aurora B^Ipl1^ is not just limited to detecting tensionless attachments [16–20]. The kinase activity of Aurora B^Ipl1^ also counteracts PP1 phosphatase recruitment to kinetochores [21]. The activity of PP1 at kinetochores is required to terminate the SAC signaling and to help cells proceed into anaphase. PP1 dephosphorylates kinetochore substrates and thus promotes the stabilization of kinetochore-MT attachments [2]. Premature recruitment of PP1 to kinetochores results in early termination of SAC signals and leads to chromosome missegregation [22].

Shugoshin, another tension sensor, also detects tensionless kinetochore-MT attachments and promotes the biorientation of sister kinetochores during mitosis. Unlike Aurora B^Ipl1^, shugoshin cannot directly destabilize tensionless attachments but acts as an adaptor protein in recruiting Aurora B^Ipl1^, thus promoting the biorientation of sister kinetochores. Though shugoshin plays an important role in the biorientation of chromosomes, several reports indicate that during mitosis, it is dispensable for the SAC activation [23–25]. Shugoshin primarily localizes to centromeres, and phosphorylation of histone H2A at T120 in humans/S121 in yeasts by the SAC component Bub1 kinase plays a central role in targeting it to centromeres [26,27].

*Cryptococcus neoformans*, a budding yeast, diverged from other ascomycete yeasts such as *S*. *cerevisiae* and *S*. *pombe* at least 500 MYA [28]. *C*. *neoformans* is an opportunistic pathogen belonging to Basidiomycota and is considered a critical priority fungal pathogen by the World Health Organization (WHO). Several species of *Cryptococcus* cause fungal meningitis [29], a fatal infection in immunocompromised patients. *C*. *neoformans* undergoes semi-open mitosis, and its nuclear division occurs in the daughter bud [30]. This is strikingly different as compared to relatively well-studied yeasts such as *S*. *cerevisiae* and *C*. *albicans* where the nuclear division occurs at the mother-daughter bud-neck junction [31]. The kinetochore architecture is significantly rewired *in C*. *neoformans*, where a linker protein bridgin presumably replaced the loss of most inner kinetochore proteins [32,33]. Moreover, metazoan-like kinetochore maturation dynamics in *C*. *neoformans* during cell cycle progression is distinctly different from most other budding yeast species [30]. The cell cycle-associated dynamics of the centromere-kinetochore complex in *C*. *neoformans* differ as well. Unlike other well-studied yeasts such as *S*. *cerevisiae*, *S*. *pombe*, *and C*. *albicans*, where kinetochores are constitutively clustered, the kinetochores in *C*. *neoformans* exhibit differential clustering dynamics, where they are unclustered during interphase and cluster during mitosis.

Despite high medical importance, mechanisms monitoring kinetochore-MT attachments have not been extensively studied in basidiomycete yeasts due to lack of molecular tools. Studies to explore the roles of SAC components Bub1 (Madbub), Mps1, and Mad1 in the checkpoint response using *C*. *neoformans* have just begun [34,35]. Moreover, *C*. *neoformans* exhibits aneuploidy-mediated fluconazole resistance [36]. Studying factors that regulate kinetochore-MT attachments in this organism with rewired kinetochore architecture and metazoan-like mitotic events might shed light on understanding aneuploidy-driven drug resistance.

Here, we probed the role of an evolutionarily conserved protein, shugoshin, in *C*. *neoformans* in monitoring the kinetochore-MT attachments during mitosis. We propose that shugoshin performs this function by maintaining higher Aurora B^Ipl1^ and lower PP1 levels at kinetochores during MT depolymerizing conditions. Unlike ascomycete yeasts, shugoshin predominantly localizes to SPBs, along spindle MTs and a minor pool at kinetochores during mitosis. Apart from the atypical localization, we provide evidence that support that shugoshin targeting to its subcellular sites is not dependent on the kinase activity of Bub1.

## Results

### Shugoshin is required for efficient metaphase arrest in response to microtubule depolymerizing drugs

**Fig 1:**
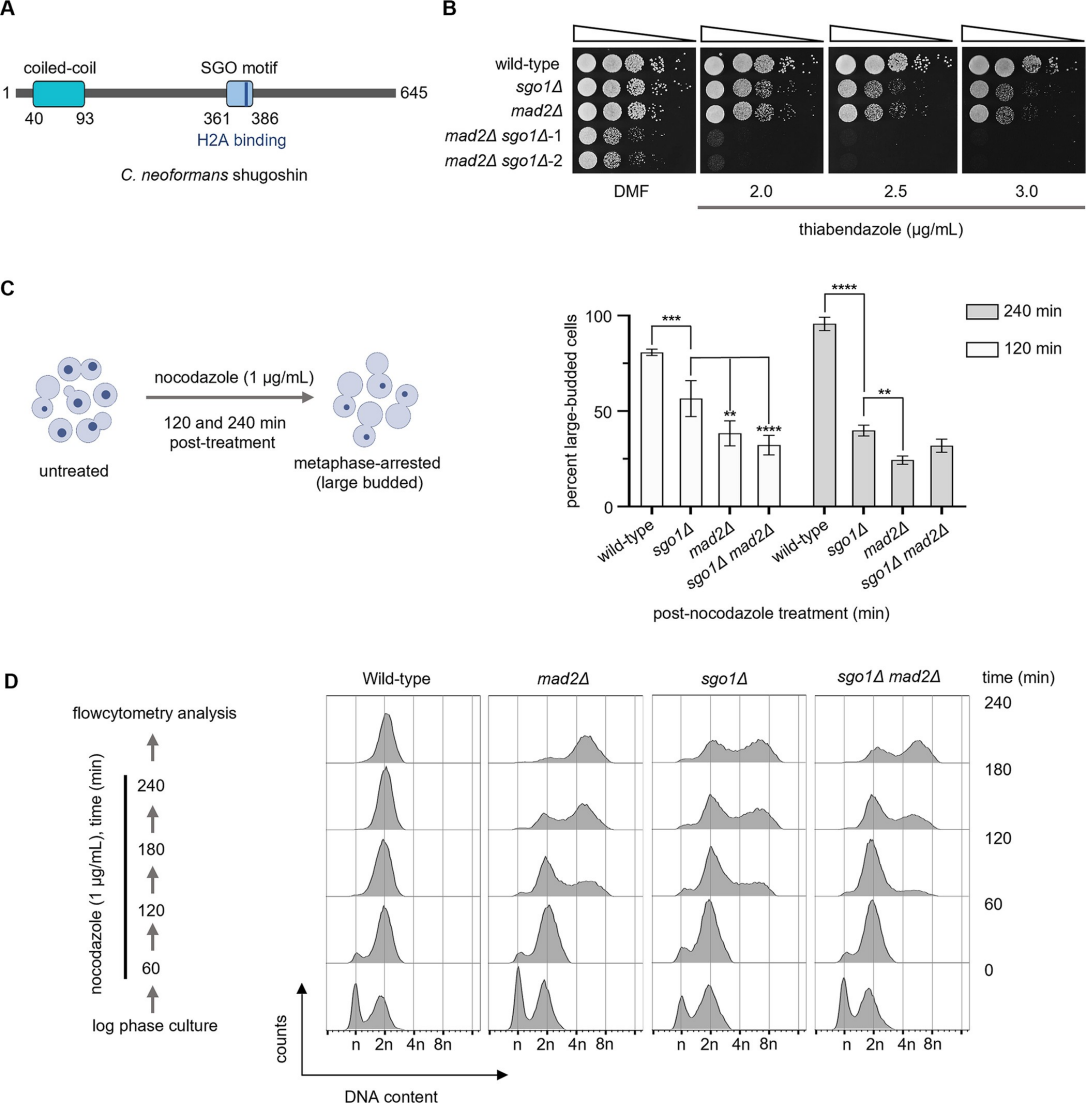
Shugoshin is required for a robust spindle assembly checkpoint arrest. **(A)** Schematic of the predicted motifs present in Sgo1 of *C*. *neoformans*. **(B)** A ten**-**fold serial dilution spotting assay to score for the sensitivity of strains CNVY108 (*SGO1 GFP-H4*), CNSD110 (*GFP-H4 sgo1Δ*), SHR741 (*GFP-H4 mad2Δ*), CNSD148 (*GFP-H4 sgo1Δ mad2Δ*) on plates containing the indicated concentrations of thiabendazole (TBZ). No drug represents DMF (dimethyl formamide) only. The plates were incubated at 30°C for 24 h. **(C)** *Left*, schematic of the assay to score for cell cycle arrest phenotypes when treated with nocodazole. Cells treated with nocodazole for 120 and 240 min were scored for cell cycle arrest phenotypes. *Right*, bar graphs representing the proportion of large-budded cells in the indicated strains, N = 3, n>100, cells counted in each experiment. Two-way ANOVA with Tukey’s multiple comparison test was used to estimate the significance. ****p<0.0001, ***p<0.001, **p = 0.0019/0.0072. Non-significant p-values were omitted from the graph. **(D)** *Left*, an outline of the assay to study the effect of nocodazole on the ploidy content of indicated strains using flow cytometry. *Right*, histograms depicting ploidy changes associated with nocodazole treatment.

*SGO1* (FungiDB ORF ID: CNAG_05516) encodes the only form of shugoshin that is expressed in *C*. *neoformans*. The putative 645-aa-long protein coded by *SGO1* has an N-terminal coiled-coil domain and a C-terminal basic SGO motif (Figs 1A, S1A and S1B). Multiple sequence alignment of amino acid sequences revealed the presence of conserved residues required for PP2A binding (S1B Fig) [37]. The SGO motif in *C*. *neoformans* shugoshin also possesses a conserved lysine residue (K382) that is required for the binding of shugoshin to phosphorylated histone H2A (S1B Fig) [26,27,38]. Previous studies reported that shugoshin plays a role in ensuring proper kinetochore-MT attachments during mitosis (see reviews [39,40]). Mutants affecting kinetochore-MT interactions and proteins participating in the error correction machinery and the SAC, are hypersensitive to MT depolymerizing drugs. To test if Sgo1 contributes to the fidelity of mitotic chromosome segregation, we created *sgo1* null mutants in *C*. *neoformans*. While Sgo1 was found to be dispensable for growth, *sgo1* null mutant cells displayed sensitivity to a MT-depolymerizing drug, thiabendazole (TBZ), similar to that of SAC mutant *mad2* (Fig 1B). These results suggest that *sgo1Δ* mutant cells in the basidiomycete yeast *C*. *neoformans* have phenotypes similar to those of ascomycete yeasts such as *Saccharomyces cerevisiae*, *Schizosaccharomyces pombe* and *Candida albicans* [23,25,41].

Next, we sought to test if enhanced sensitivity to the MT poison observed in *sgo1Δ* mutant cells accentuate when combined with *mad2Δ* mutant. To test this, we created *sgo1Δ mad2Δ* double mutants. Such double mutant cells exhibited synthetic lethality in the presence of TBZ (Fig 1B), indicating that both Sgo1 and Mad2 are required for an efficient SAC response in MT-depolymerizing conditions. This striking synthetic lethality observed suggests that shugoshin is involved in functions such as biorientation apart from promoting the SAC response, which is lost in *mad2Δ*. If shugoshin is required for an efficient SAC response in *C*. *neoformans*, it is expected that the *sgo1Δ* mutant, when combined with a kinetochore mutant, would enhance sensitivities to TBZ. To explore this possibility, we used null mutant cells of a recently reported basidiomycete-specific kinetochore linker protein bridgin (Bgi1). It has been shown that *bgi1Δ* mutants are viable but exhibit gross chromosomal missegregation [33]. *bgi1Δ* mutants, combined with *mad2Δ*, displayed enhanced sensitivity to TBZ compared to each of the single mutants alone [33]. The spotting assay indicated that *sgo1Δ bgi1Δ* double mutant cells are hypersensitive to TBZ, similar to that of *mad2Δ bgi1Δ* (S2 Fig). Together, these results reveal that shugoshin plays a role in proper kinetochore-MT interactions and/or monitors defective attachments in response to spindle poison.

MT-depolymerizing drugs arrest wild-type cells at metaphase in the large-budded stage with an undivided nucleus. Only when the defects are rectified will such cells proceed to complete the cell division. To verify whether *sgo1Δ* mutants can induce metaphase arrest when MTs are depolymerized, we performed a microscopy-based assay to score for the cell cycle defects associated with *sgo1Δ* mutants by treating cells with nocodazole (Fig 1C). Nocodazole-treated wild-type cells of *C*. *neoformans* arrest at the large-budded stage with a visibly condensed chromatin mass (Fig 1C, right). After 240 min of nocodazole treatment, >90% of wild-type cells were at the large-budded stage with a condensed undivided nucleus (Figs 1C and S1C–S1E). As expected, *mad2Δ*, a bonafide checkpoint-defective mutant, failed to arrest cells at the large-budded stage (Fig 1C, right). In the *sgo1Δ* mutants, though we initially observed an increase in the proportion of large-budded cells compared to *mad2Δ* mutants, after 240 min post-nocodazole treatment, the large-budded cell population significantly reduced (Fig 1C, right). Moreover, unlike wild-type, most large-budded cells in *sgo1Δ* mutants had an uncondensed chromatin mass similar to *mad2Δ* mutant cells (S1C–S1E Fig). In the case of *sgo1Δ mad2Δ* double mutants, no significant differences in the proportion of large-budded cells were observed when compared to *sgo1Δ* and *mad2Δ* single mutants (Fig 1C, right). Estimating the ploidy content in nocodazole-treated cells using flow cytometry (Fig 1D) revealed that wild-type cells arrested at 2n ploidy level. On the other hand, *mad2Δ* cells shifted towards 4n, indicating a failure in arresting cells at metaphase and initiating the next cell cycle events. Unlike *mad2Δ*, *sgo1Δ* cells displayed a ploidy content of 2n and 4n, revealing the presence of cells that are delayed at metaphase and cells that have initiated the next round of DNA replication. Similar results were also obtained in *sgo1Δ mad2Δ* double mutants. Taken together, we show that shugoshin is required for an efficient metaphase arrest in response to unattached kinetochores induced by MT-depolymerizing drugs.

### *sgo1Δ* mutant cells fail to maintain the SAC component Bub1 at kinetochores in response to prolonged microtubule depolymerization

**Fig 2:**
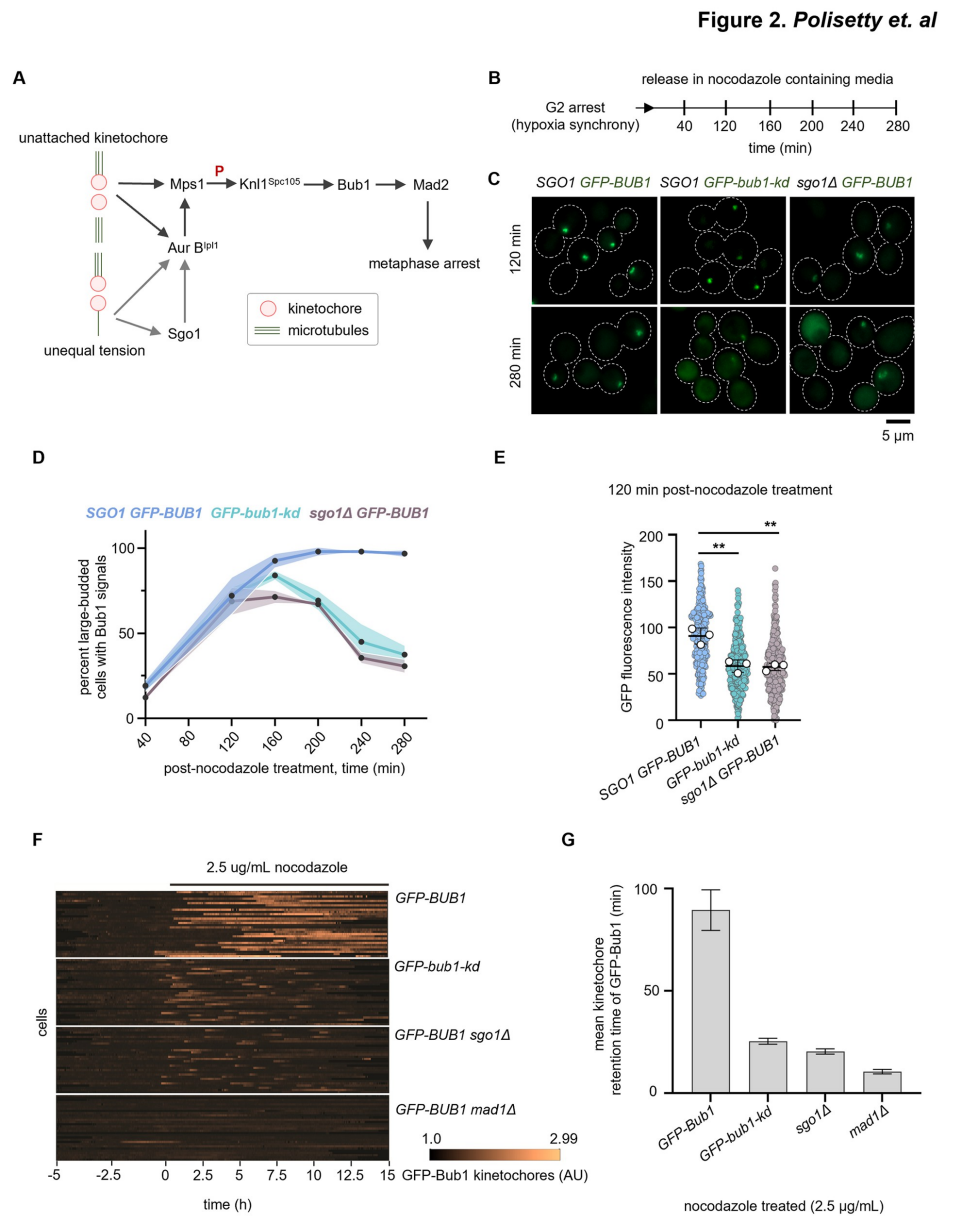
Shugoshin ensures Bub1 is retained at kinetochores to maintain the spindle assembly checkpoint arrest. **(A)** Schematic of the SAC pathway and the evolutionarily conserved role of Sgo1 and Aurora B^Ipl1^ in detecting erroneous kinetochore-MT attachments. **(B)** An experimental workflow followed to determine the GFP-Bub1 localization dynamics under nocodazole-treated conditions. **(C)** Sub-cellular localization of GFP-Bub1 localization in IL102 (*SGO1*), IL143 (*bub1-kd*) and CNSD176 (*sgo1Δ*). For representation, cells treated with nocodazole for 120 and 280 min were shown. Scale bar, 5 μm. **(D)** Quantification of the proportion of large-budded cells with GFP-Bub1 signal in indicated strains treated with nocodazole. N = 3, n>100 cells were analyzed, and the shaded regions above and below the line graphs represent error bands corresponding to SD. **(E)** Quantification of GFP-Bub1 fluorescence intensity values at 120 min post-nocodazole treatment. N = 3, n>100 cells analyzed for each experiment. White circles represent the average intensity values of replicates. A mean of the average intensity values of replicates is represented as a black bar, and error bars represent SD. An unpaired t-test was used to test the significance, ** p = 0.0092, ns = 0.1131 **(F)** Microfluidics assays to determine the retention time of GFP-Bub1 at kinetochores in response to nocodazole treatment. Temporal heat maps of 30 randomly selected cells are shown. The heat map represents the changes in kinetochore localization signal of GFP-Bub1 over time. Each bright track on the *y*-axis of the heat map represents GFP-Bub1 signals from an individual cell (median of the brightest 5 pixels in each cell divided by the overall cell median brightness). The length of each bright track along the *x*-axis represents the time (min). The time of addition of nocodazole (2.5 μg/mL) is considered as 0 h. Images are taken every 2 min for 15 h. Assays were performed using strains IL102 (*GFP-BUB1*), IL143 (*GFP-bub1-kd*), CNSD176 (*GFP-BUB1 sgo1Δ*), and IL089 (*GFP-BUB1 mad1Δ*) strains. **(G)** Bar graphs representing the quantitative analysis of the GFP-Bub1 retention time at kinetochores obtained from microfluidics assays in the above-indicated strains. n = 30, error bars represent SEM.

Why are *sgo1Δ* mutant cells not arrested when treated with an MT poison at metaphase? It is possible that *sgo1Δ* mutant cells arrest at metaphase immediately after being treated with the MT poison but were unable to maintain the arrest upon prolonged exposure to the drug. To test this, we localized the SAC component Bub1 (Fig 2A) tagged with GFP in wild-type and *sgo1Δ* cells. A recent report [34] suggested that Bub1 localizes to kinetochores in *C*. *neoformans* and plays a central role in the SAC-mediated arrest. We synchronized *C*. *neoformans* cells by arresting them at G2 using hypoxia [42] and released them in the presence of nocodazole (refer to Materials and Methods for further details) (Fig 2B). Inducing physical hypoxia in *C*. *neoformans* results in ’unbudded’ G2 arrest. These G2-arrested cells enter mitosis synchronously when subjected to extensive aeration. Upon release into nocodazole-containing media, wild-type cells accumulated at the large-budded stage (S3 Fig) with GFP-Bub1 signals, which were maintained until the end of the experiment (Fig 2C and 2D). In the case of *sgo1Δ* cells, the proportion of Bub1-positive large-budded cells increased similarly to that of wild-type until 120 min. However, beyond 200 min post-nocodazole treatment, a sudden decline in the proportion of Bub1-positive large-budded cells was observed in the mutant (Fig 2C and 2D). Studies in *S*. *pombe* have shown that the kinase-dead version of Bub1 is checkpoint-proficient but fails to maintain prolonged SAC arrest [27]. A similar observation was reported in *C*. *neoformans*, where *bub1-kd* mutant cells failed to sustain the arrest induced by nocodazole [34]. To understand how checkpoint responses differ in *sgo1Δ* from *bub1-kd*, we compared GFP-Bub1 localization dynamics in hypoxia-synchronized cells treated with nocodazole (Fig 2C and 2D). Though neither *sgo1Δ* nor *bub1-kd* mutant cells could maintain the arrest like the wild-type, *bub1-kd* cells were able to maintain the arrest longer than *sgo1Δ* cells.

Moreover, Bub1 signal intensities in *sgo1Δ* and *bub1-kd* mutants were significantly reduced compared to the wild-type (Fig 2E). To further support these observations, we resorted to a single-cell microfluidics assay to quantitatively measure the checkpoint response in individual cells treated with the MT poison (Fig 2F and 2G). Using this system, we probed the GFP-Bub1 dynamics in wild-type, *sgo1Δ*, *bub1-kd*, *sgo1Δ bub1-kd*, and *mad1Δ* mutant cells treated with nocodazole. Similar to the results observed in the experiment performed with the synchronized cells, neither *sgo1Δ* nor *bub1-kd* mutant cells could maintain checkpoint arrest like wild-type as indicated by the lower mean kinetochore residence times of Bub1 (Fig 2G). Checkpoint defective *mad1Δ* mutant cells were used as a control. The *sgo1Δ bub1-kd* double mutant behaved like the *sgo1Δ* single mutant (S4A and S4B Fig). These results reveal that *sgo1Δ* cells, similar to *bub1-kd* cells, are proficient in checkpoint activation but cannot maintain checkpoint signals during prolonged treatment with nocodazole.

### Aurora B^Ipl1^ maintenance at kinetochores is affected in *sgo1Δ* mutants during prolonged checkpoint arrest

**Fig 3:**
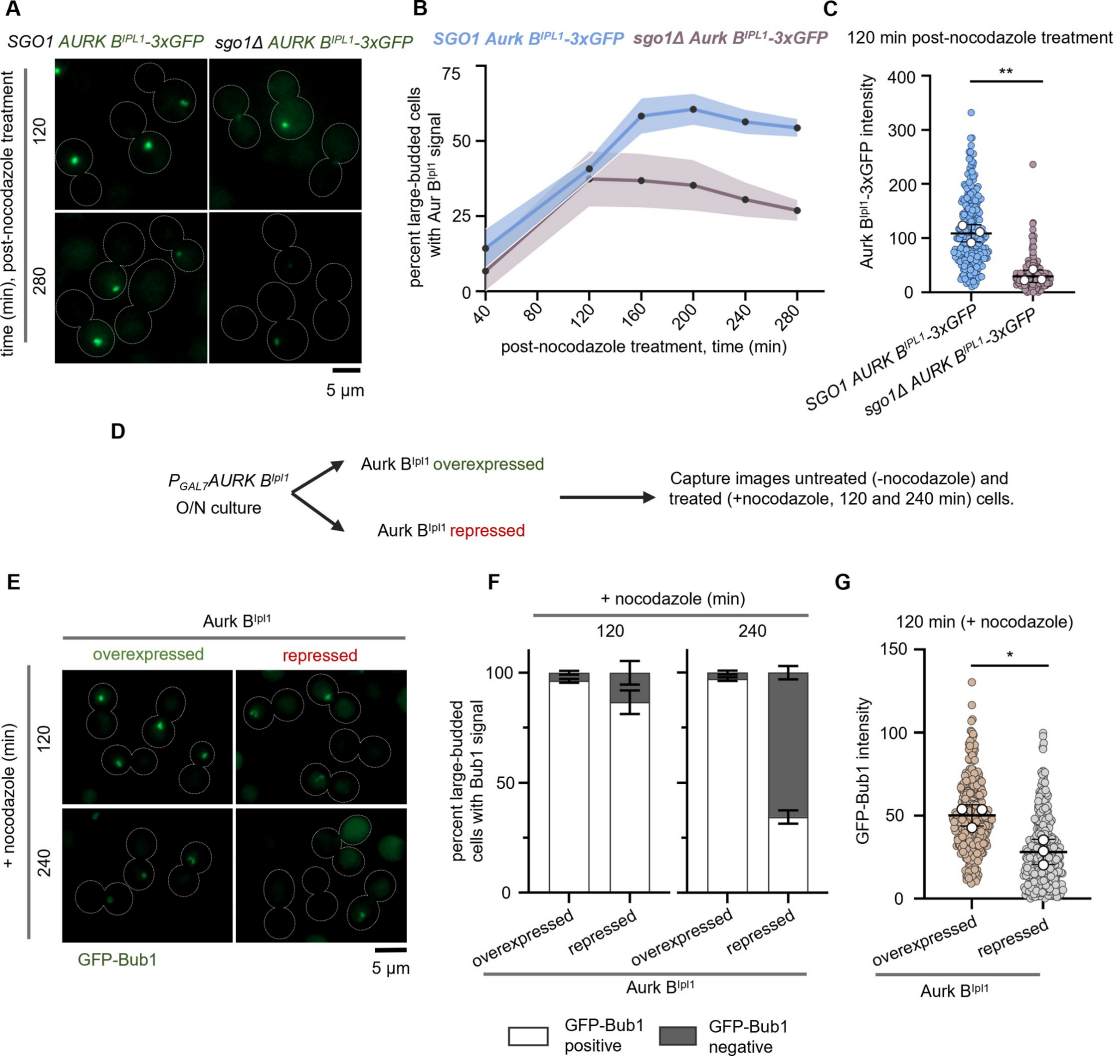
Shugoshin-dependent Aurora B^Ipl1^ pool at kinetochores ensures maintenance of Bub1 to prolong the checkpoint arrest. **(A)** Sub-cellular localization of Aurora B^Ipl1^-3xGFP localization in CNSD190 (*AURORA B*^*Ipl1*^*-3xGFP mCherry-CENP-A SGO1*) and CNSD196 (*AURORA B*^*Ipl1*^*-3xGFP mCherry-CENP-A sgo1Δ*) strains treated with nocodazole. For representation, cells treated with nocodazole for 120 and 280 min were shown. Scale bar, 5μm. **(B)** Quantification of the proportion of large-budded cells with Aurora B^Ipl1^-3xGFP signals in the indicated strains. N = 3, n>100 cells were analyzed, and the shaded regions above and below the line graph represent error bands corresponding to SD. **(C)** Quantification of Aurora B^Ipl1^-3xGFP fluorescence intensity values at 120 min post-nocodazole treatment. N = 3, n>58 cells for each experiment. White circles represent the average intensity values of replicates. The mean of the average intensity values of replicates is represented as a black bar. Error bars represent SD. An unpaired t-test was used to test the significance, ** p = 0.0013. **(D)** Schematic to assess the response of Aurora B^Ipl1^ (over)expression or depletion to nocodazole treatment. **(E)** Sub-cellular localization of GFP-Bub1 localization in CNSD205 (*GAL7-3xFLAG-AURORA B*^*IPL1*^ *GFP-BUB1*) Aurora B^Ipl1^ (over)expressed and repressed conditions treated with nocodazole. Cells treated with nocodazole for 120 and 240 min are shown for representation. Scale bar, 5 μm. **(F)** Bar graphs representing the proportion of large-budded cells with or without of GFP-Bub1 signals at 120 and 240 min of nocodazole treatment. N = 3, n>100 cells analyzed for each experiment. Error bars represent SD. **(G)** Quantification of GFP-Bub1 fluorescence intensity values in Aurora B^Ipl1^ (over)expressed and repressed conditions at 120 min of nocodazole treatment. White circles represent the average intensity values of replicates. The mean of the average intensity values of the replicates is represented as a black bar. Error bars represent SD. N = 3, n> 79 cells analyzed for each experiment. An unpaired t-test was used to test the significance, *p = 0.0439.

Shugoshin interacts with CPC proteins and helps in either the recruitment or maintenance of CPC components at centromeres/kinetochores in *S*. *cerevisiae*, *S*. *pombe* and humans [40,43]. Previously, we established the role of Aurora B^Ipl1^ (a CPC component) in regulating nuclear division during mitosis in *C*. *neoformans* [44]. Although it has been shown that the entry of Aurora B^Ipl1^ into the nucleus coincides with the nuclear envelope breakdown (NEBD), its localization to kinetochores remained unexplored. In this study, we colocalized Aurora B^Ipl1^ with the inner kinetochore protein CENP-A at different cell cycle stages (S5A Fig). During G2, when kinetochores are clustered, we could only detect weak signals of Aurora B^Ipl1^ that intensified as cells entered metaphase. Apart from the kinetochore localization, we could also observe spindle-like localization of Aurora B^Ipl1^ during anaphase (S5A Fig). Upon treatment with nocodazole, we observed colocalization of Aurora B^Ipl1^ with kinetochores (S5B Fig). To test whether kinetochore localization of Aurora B^Ipl1^ is affected in *sgo1Δ* cells in *C*. *neoformans*, we synchronized both wild-type and *sgo1Δ* mutant cells by inducing physical hypoxia followed by a release in nocodazole-containing media (Fig 2B). We scored for Aurora B^Ipl1^-3xGFP-positive large-budded cells. In *sgo1Δ*, we observed a reduced proportion of Aurora B^Ipl1^-positive large-budded cells over time compared to wild-type (Figs 3A, 3B and S6). Similar to the Bub1 levels in *sgo1Δ* mutant cells, a reduction in the intensity of the Aurora B^Ipl1^ signals in the *sgo1Δ* cells was also observed compared to the wild-type (Fig 3C). Taken together, we conclude that Sgo1 is required to maintain Bub1 and Aurora B^Ipl1^ at kinetochores when MTs are depolymerized.

### Aurora B^Ipl1^ is required for the maintenance of Bub1 at kinetochores in response to microtubule depolymerization

Reduced Aurora B^Ipl1^ levels at kinetochores in *sgo1Δ* cells prompted us to test whether the absence of Aurora B^Ipl1^ leads to failure of checkpoint arrest when treated with MT depolymerizing agents. For conditional depletion of Aurora B^Ipl1^, we placed the gene under the *GAL7* promoter. We treated Aurora B^Ipl1^ –(over)expressed (permissive, galactose media) and Aurora B^Ipl1^-depleted (non-permissive, glucose media) cells with nocodazole (Fig 3D). We assayed for large-budded cells with a condensed chromatin mass as observed at metaphase. Cells (over)expressing Aurora B^Ipl1^ and treated with nocodazole displayed a higher proportion of large bud arrested cells. In contrast, cells depleted of Aurora B^Ipl1^ failed to arrest at the large-budded stage and exhibited an increase in the proportion of tri-budded cells (S7A and S7B Fig). Moreover, approximately 50% of the large-budded cells displayed an uncondensed nuclear mass, similar to what was observed in *mad2Δ* mutants (S7A and S7B Fig). These results suggest that Aurora B^Ipl1^ is required for an efficient metaphase arrest when cells are treated with a spindle poison in *C*. *neoformans*.

To confirm these observations further, we examined GFP-Bub1 localization dynamics in cells depleted of Aurora B^Ipl1^ and treated with nocodazole (Fig 3E). Aurora B^Ipl1^-depleted cells subjected to nocodazole treatment for 120 and 240 min were able to load Bub1 initially but failed to maintain Bub1 at kinetochores upon prolonged treatment with nocodazole (Fig 3E and 3F), similar to what was observed in *sgo1Δ* mutant cells. We also noticed a significant reduction in the levels of Bub1 at kinetochores in Aurora B^Ipl1^-depleted cells similar to that of *sgo1Δ* mutants (Fig 3G). These results reveal that Aurora B^Ipl1^ in *C*. *neoformans* helps maintain Bub1 at kinetochores in the presence of nocodazole.

### Shugoshin antagonizes the recruitment of PP1 at kinetochores in response to nocodazole treatment

**Fig 4:**
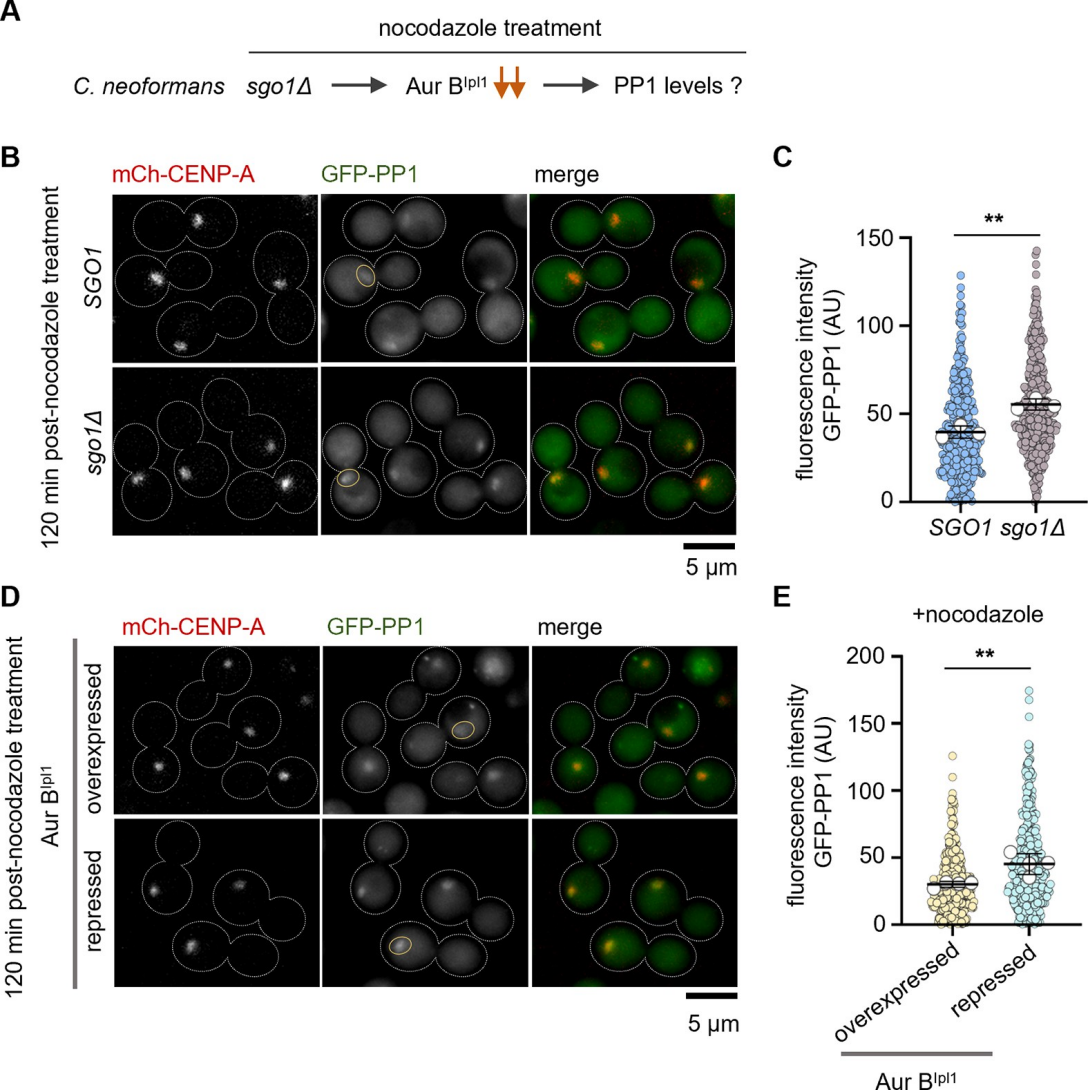
Sgo1-mediated Aurora B^Ipl1^ recruitment prevents excess PP1 phosphatase at the kinetochore. **(A)** The effect of *sgo1Δ* on maintaining a balance between Aurora B^Ipl1^ and PP1 levels at kinetochore in *C*. *neoformans*. **(B)** Sub-cellular localization of CNKB003 (*GFP-PP1 mCherry-CENP-A SGO1*) and CNSD180 (*GFP-PP1 mCherry-CENP-A sgo1Δ*) cells treated with nocodzole (1 μg/mL) for 120 min. Yellow ovals highlight PP1-GFP signals. **(C)** Quantification of GFP-PP1 fluorescence signal intensity values in the indicated strains treated with nocodazole. N = 3, n>100 cells were analyzed for each experiment. White circles represent the average GFP-PP1 signal intensities calculated for each experimental repeat. An unpaired t-test was used to determine statistical significance. **p = 0.0044. The mean of the average intensity values of replicates is represented as a black bar. Error bars represent SD. **(D)** Microscopic images of CNKB023 (*GFP-PP1 mCherry-CENP-A GAL7-3xFLAG AURORA B*^*IPL1*^) Aurora B^Ipl1^ (over)expressed and repressed conditions treated with nocodazole (1 μg/mL) for 120 min. **(E)** Quantification of fluorescence intensity values of GFP-PP1 signals in conditions mentioned above. N = 4, n = 100 cells for each experiment. White circles represent the average intensity values of replicates. Error bars represent SD. The mean of the average intensity values plotted is represented as a black bar. Paired t-test was used to determine statistical significance. **p = 0.0069.

The kinase activity of Aurora B^Ipl1^ and the phosphatase activity of PP1 are antagonistic at kinetochores [45,46]. We hypothesized that a reduction in Aurora B^Ipl1^ levels leads to increased PP1 levels (Fig 4A), and higher PP1 levels may lead to the loss of Bub1 from kinetochores. We expressed PP1-GFP (FungiDB ORF ID CNAG_03706; S8 Fig) in a CENP-A-mCherry expressing strain (CNKB003). First, we studied the effect of *sgo1Δ* on PP1 localization during nocodazole treatment (Fig 4B). Both wild-type and *sgo1Δ* cells exhibited GFP-PP1 signals at kinetochores during the metaphase-arrested stage. Quantifying the fluorescence signal intensity of PP1 revealed a marginal but significant increase in the GFP-PP1 signal intensities at kinetochores in *sgo1Δ* compared to wild-type cells (Fig 4C). A similar result was observed when Aurora B^Ipl1^-depleted cells were treated with nocodazole (Fig 4D and 4E). The SAC can be silenced by dephosphorylation of MELT repeats on Knl1^Spc105^ with the help of PP1. As a result, checkpoint components are removed from kinetochores [20,47–51]. Premature recruitment of PP1 to kinetochores is detrimental and leads to chromosome mis-segregation [22]. Based on the results described above, we propose that the increase in PP1 levels at kinetochores leads to the loss of Bub1 signals in the absence of shugoshin or Aurora B^Ipl1^, which interferes with the SAC maintenance in MT depolymerizing conditions.

### The tension-sensing function of shugoshin is conserved in *C*. *neoformans*

Shugoshin senses tensionless attachments by recruiting several factors that play a role in correcting tensionless attachments [40]. Cohesin is a molecular glue that holds two sister chromatids together during mitosis until metaphase. Cohesin plays an essential role in generating tension that resists the pulling force of MTs, aiding high-fidelity chromosome segregation [52,53]. Loss of cohesion at centromeres results in tensionless kinetochore-MT attachments. To test if Sgo1 in *C*. *neoformans* detects tensionless attachments, we depleted one of the cohesin subunits, Scc1/Mcd1/Rad21, by placing it under the *GAL7* promoter both in the wild-type and *sgo1Δ* mutant cells. *C*. *neoformans* Scc1 (FungiDB ORF ID CNAG_01023) harbors N- and C-terminal Scc1 domains (S9A and S9B Fig). We show that like *S*. *cerevisiae* and *S*. *pombe*, *SCC1* in *C*. *neoformans* is essential for viability (S9C Fig). Upon depletion of Scc1, a high proportion of wild-type cells are arrested at the large-budded stage (S9D–S9F Fig). In contrast, the *sgo1Δ* mutants depleted of Scc1 did not show such an increase in the proportion of large-budded cells, indicating that shugoshin plays a role in detecting tensionless kinetochore-MT attachments and delays the cell cycle progression.

### Shugoshin is enriched at the spindle pole bodies (SPBs) and spindle during mitosis

**Fig 5:**
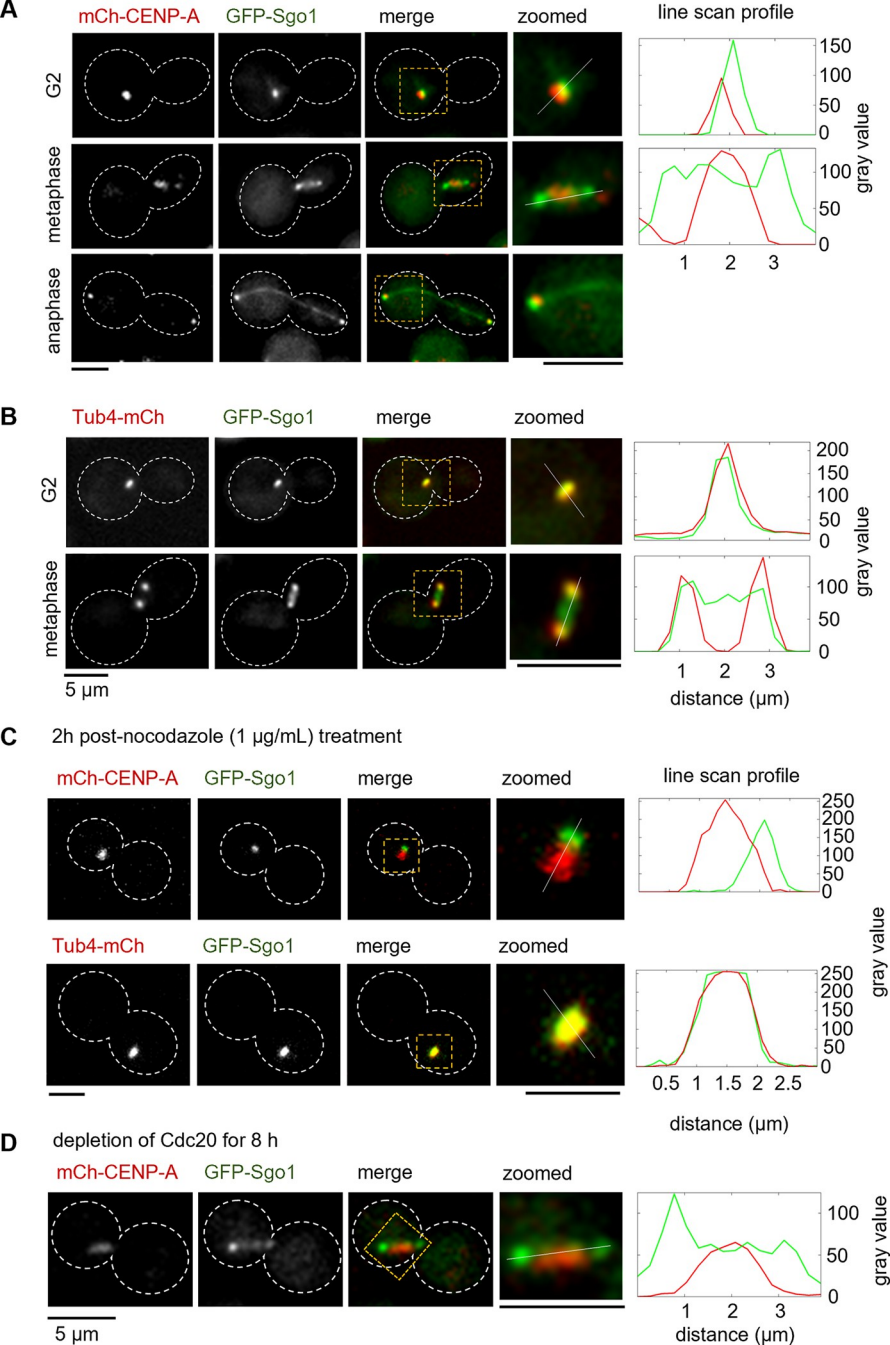
Kinetochore proximal association of Sgo1 in MT-depolymerizing conditions. **(A-D)** *Left*, microscopic images of GFP-Sgo1 localized with a kinetochore marker, CENP-A, and an SPB marker, Tub4, at different stages of the mitotic cell cycle, in nocodazole treated and Cdc20 depleted conditions. Scale bar, 5 μm. **(A)** CNSD125 (*GFP-SGO1 mCherry-CENP-A*). **(B)** CNSD130 (*GFP-SGO1 TUB4-mCherry*). **(C)** Nocodazole (1 μg/mL) treated condition. *Top panel*, CNSD125. *Bottom panel*, CNSD130. **(D)** Cdc20 depletion condition, CNSD167 (*GFP-SGO1 mCherry-CENP-A GAL7-3xFLAG-CDC20*). *Right*, line scan profiles of both GFP (Sgo1) and mCherry (CENP-A, Tub4) fluorescence signals. Distance plotted on the *x*-axis represents the length of the line drawn on the image to measure fluorescence intensity. Gray values on the *y*-axis represent the fluorescence signal intensities.

Thus far, we reveal an unknown role of Sgo1 in maintaining Bub1 through the balance of Aurora B^Ipl1^ and PP1 at kinetochores in *C*. *neoformans*. Next, we examined the cell cycle localization dynamics of Sgo1 in *C*. *neoformans*. Shugoshin localizes to the kinetochore during cell division in most species studied [40] (S11 Fig). To find the sub-cellular localization of Sgo1, we expressed GFP-Sgo1 from its native locus in the CENP-A-mCherry tagged strain. We synchronized the cells using hypoxia to examine the cell cycle stage-specific localization of Sgo1. No signals of Sgo1 could be observed during most of the interphase. In G2, where all centromeres cluster as a single punctum [30,33], we observed Sgo1 signals near clustered centromeres (Fig 5A). Upon entry into mitosis, Sgo1 appeared as two distinct puncta that flanked the bar-like signals of CENP-A. Apart from the punctate signals of Sgo1, we observed weak signals along the pole-to-pole axis during metaphase and anaphase that resembled spindle-like localization. To examine whether Sgo1 localizes to spindle pole bodies (SPBs), we expressed Tub4-mCherry (γ-tubulin) in the GFP-Sgo1 expressing strain. GFP-Sgo1 signals colocalized with SPBs during G2 and mitotic stages (Fig 5B), indicating that Sgo1 localizes predominantly to SPBs during the mitosis in *C*. *neoformans*.

To test if the spindle-like localization during mitosis depends on the spindle MT integrity, we arrested cells at metaphase using two methods. First, we used nocodazole to depolymerize MTs that activate the SAC and arrest cells at metaphase. Second, we depleted Cdc20, an anaphase-promoting complex/cyclosome (APC/C) component. Depletion or inactivation of Cdc20 leads to metaphase arrest in *S*. *cerevisiae* [54]. Upon treating *C*. *neoformans* cells with nocodazole, we observed that Sgo1 colocalized with the SPB (Fig 5C). On the other hand, depletion of Cdc20 by placing it under the galactose regulatable promoter (*GAL7*) resulted in metaphase arrest, and the spindle-like localization of shugoshin along the pole-to-pole axis was maintained (Fig 5D). These observations led us to conclude that the spindle-like localization of Sgo1 requires intact MTs during mitosis.

### The kinase activity of Bub1 is dispensable for the function and localization of shugoshin during mitosis in *C*. *neoformans*

**Fig 6:**
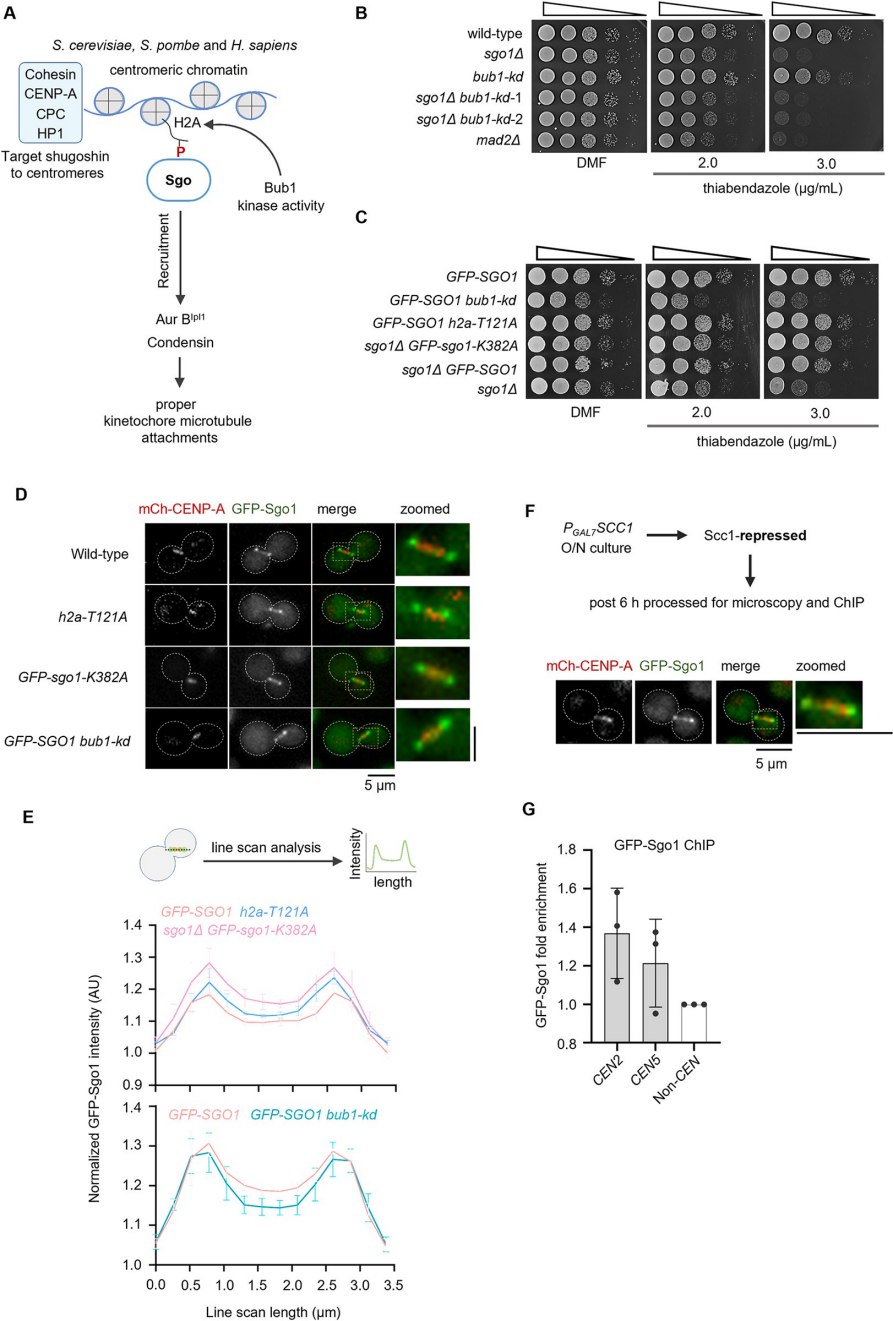
The kinase activity of Bub1 is dispensable for shugoshin-mediated maintenance of the spindle assembly checkpoint. **(A)** Schematic of the conserved pathway of shugoshin recruitment to kinetochores. **(B)** A ten-fold serial dilution spotting assay to score for the sensitivity of the CNSD110 (*sgo1Δ*), IL143 (*GFP-bub1-kd*), CNSD173 (*sgo1Δ GFP-bub1-kd*) double mutants and SHR866 (*mad2Δ*) to thiabendazole. **(C)** A ten-fold serial dilution spotting assay to score for the sensitivity of the CNSD125 (*GFP-SGO1 mCherry-CENP-A*), CNSD215 (*GFP-SGO1 mCherry-CENP-A bub1-kd-3XFLAG*), CNSD219 (*GFP-SGO1 mCherry-CENP-A h2a-T121A*), CNSD218 (*sgo1Δ GFP-sgo1-K382A-SH1 mCherry-CENP-A*), CNSD207 (*sgo1Δ GFP-SGO1-SH1*) and CNSD110 (*sgo1Δ*). **(D)** Microscopic images of GFP-Sgo1 localized with CENP-A at metaphase stage in wild-type and mutant backgrounds. Scale bar, 5 μm. **(E)** *Top*, Schematic showing the line scan analysis method adopted to score for the fluorescence intensity of GFP-Sgo1 along the spindle and SPBs. *Middle*, line scan profile of GFP-Sgo1 in the indicated strains synchronized using thiabendazole (10 μg/mL) treatment. *Bottom*, line scan profile of GFP-Sgo1 in the indicated strains synchronized using hypoxia. Distance on the *x*-axis represents the length of the line drawn on the image to measure fluorescence intensity. The *y*-axis represents the fluorescence signal intensity values. N = 3, n>20 cells were analysed for each experiment. Each line graph represents mean fluorescence intensity values calculated from three experimental repeats and error bars represent SD. **(F)** *Top*, Schematic of the assay used to arrest cells at metaphase by depleting Scc1. *Below*, microscopic images of GFP-Sgo1 localized with CENP-A metaphase in Scc1 depleted conditions. **(G)** Fold enrichment of Sgo1 at kinetochores in metaphase arrested cell obtained by cross-linking chromatin immunoprecipitation (ChIP) followed by quantitative polymerase chain reaction (qPCR). *CEN2* and *CEN5* represent centromeres 2 and 5, non-CEN region represents *LEU2* locus. N = 3, and error bars represent SD.

Studies from several yeast species and humans reveal that Bub1 is one of the major factors that recruit shugoshin to kinetochores. Although Bub1 plays a critical role, several other proteins such as cohesin, heterochromatin protein (HP1), CPC, and CENP-A also contribute to the targeting of shugoshin to kinetochores, see review [40] (Fig 6A). In the present study, to test if the Bub1 kinase activity required for shugoshin’s function is evolutionarily conserved in *C*. *neoformans*, we utilized the kinase-dead (*bub1-kd*) mutant of Bub1. *In vitro* assays reported earlier [34] revealed that Bub1 could phosphorylate the reconstituted human H2A containing nucleosomes, and kinase-dead mutants of Bub1 fail to phosphorylate. We performed a genetic interaction assay by scoring the TBZ sensitivity of *sgo1Δ*, *bub1-kd*, and *sgo1Δ bub1-kd* mutants (Fig 6B). If both Sgo1 and Bub1 kinase activity function in the same pathway in *C*. *neoformans*, one would expect little to no change in the sensitivity of double mutants compared to either of the single mutants, *sgo1Δ* and *bub1-kd*. By dilution spotting assay, we observed that the *bub1-kd sgo1Δ* double mutant cells behaved like that of the *sgo1Δ* mutant (Fig 6B). Moreover, the sensitivity of *sgo1Δ* mutant cells to TBZ is higher than that of *bub1-kd* mutant. There are two possibilities for the above observations. First, the enhanced sensitivity of the *sgo1Δ* mutant could be due to the additional functions of the shugoshin that do not rely on the kinase activity of Bub1. Second, the kinase activity of Bub1 does not play a significant role in dictating the kinetochore/kinetochore proximal localization of Sgo1. To test these possibilities, we have localized GFP-Sgo1 and performed a genetic interaction assay using mutants known to disrupt Bub1 kinase-mediated targeting of shugoshin in other species.

The lysine residue K382 in Sgo1 in *C*. *neoformans* corresponds to the lysine K492 in Sgo1 in humans (S1B Fig). This residue is known to play a role in binding to phosphorylated H2A [26,38]. A mutation in the lysine residue of shugoshin in fission yeast (K298) or humans (K492) results in the loss of kinetochore localization of shugoshin in mitosis [26,38]. Similarly, mutating the S121 (budding/fission yeast) or T121 (humans) residue of histone H2A also abrogates the kinetochore localization of the shugoshin [26,38]. To test the role of these conserved residues in *C*. *neoformans*, we mutated K382 in Sgo1 (S1B Fig) and T121 in H2A to alanine (S10A Fig). The plasmid constructs harboring either wild-type *SGO1* or a *sgo1*-*K382A* allele were integrated into the *SAFE HAVEN* locus (chromosome 1) [55] of the *sgo1Δ* strain. An overlap PCR construct harboring the *h2a-T121A* mutation was transformed into a strain expressing GFP-SGO1 and mCherry-CENP-A. If the kinase activity of Bub1 is indeed dispensable for shugoshin’s checkpoint function during mitosis, the strain carrying the *GFP-sgo1-K382A* allele would rescue the sensitivity to spindle poisons. Similarly, strain harboring the *h2a-T121A* allele would not show any sensitivity. Spotting assays on TBZ plates revealed that both sgo1*-K382A* and *h2a-T121A* mutants behaved like wild-type cells (Fig 6C). To further confirm these results, we localized Sgo1 in the above-mentioned mutant strains. Since Sgo1 exhibits both SPB and spindle-like localization during metaphase, we have obtained metaphase cells using hypoxia or thiabendazole arrest followed by release to score for the localization (refer to materials and methods for details). In both budding and fission yeasts, *bub1-kd*, *h2a-SA*, and *sgo-KA* mutants abolish kinetochore localization of shugoshin [26]. In sharp contrast, we observed that *sgo1-K382A*, *h2a-T121A*, and *bub1-kd* mutants in *C*. *neoformans* did not abolish the SPB or spindle-like localization of Sgo1 during metaphase (Fig 6D and 6E). Sgo1 levels were unaffected in the above mutants (S10B Fig). These results confirm that the kinase activity of Bub1 is dispensable for the recruitment of shugoshin and its SAC maintenance function in *C*. *neoformans*.

In *C*. *neoformans*, Sgo1 predominantly localizes to SPBs and the spindle during metaphase. The metaphase spindle localization of Sgo1 overlaps with CENP-A (Fig 5A). To probe if the kinetochores at the metaphase stage bind to Sgo1, we performed chromatin immunoprecipitation (ChIP) assay in Scc1-depleted cells (Fig 6F, top panel). Microscopy analysis of Scc1-depleted cells showed both SPB and spindle-like localization of Sgo1 (Fig 6F, bottom panel) as observed previously (Fig 5). ChIP assay indicates that there is only a minor enrichment of Sgo1 at centromeres in *C*. *neoformans* (Fig 6G), suggesting that the association of Sgo1 with centromeres could be transient in nature and/or only a minor pool of Sgo1 is associated with kinetochores and the majority could be along the spindle.

## Discussion

In this study, we characterized the mitotic functions of shugoshin in *C*. *neoformans*. The only form of shugoshin, coded by *SGO1*, predominantly localizes to SPBs and along the mitotic spindle in *C*. *neoformans*. In response to prolonged MT depolymerization, Sgo1 maintains adequate Aurora B^Ipl1^ levels at kinetochores to antagonize premature phosphatase action of PP1 at kinetochores. Thus, by regulating an optimum balance of the protein kinase Aurora B^Ipl1^ and the protein phosphatase PP1, shugoshin retains checkpoint responses until errors are corrected. Together, we report that Sgo1 regulates PP1 levels at kinetochores in response to microtubule depolymerization. We also demonstrated that the checkpoint maintenance function of Sgo1 is independent of the evolutionarily conserved Bub1 kinase activity-mediated targeting of Sgo1 to centromeres/kinetochores.

Sgo1 is not required to activate the SAC; rather, it helps prolong the SAC response by maintaining the levels of Bub1 at kinetochores in *C*. *neoformans*. Similarly, Aurora B^Ipl1^ also helps in the maintenance of Bub1 levels at kinetochores in response to MT depolymerization conditions. Studies in *S*. *cerevisiae*, *S*. *pombe*, flies, and humans reveal that shugoshin interacts with the chromosome passenger complex (CPC) and is required for either recruitment or maintenance of Aurora B^Ipl1^ to correct tensionless kinetochore attachments [40,43]. We show that apart from maintaining Bub1 levels at kinetochores, Sgo1 is also required to maintain Aurora B^Ipl1^ levels when treated with nocodazole. Microfluidics and time course assays (Figs 2D, 2F and 3B) indicate that *sgo1Δ* mutants can load Bub1 or Aurora B^Ipl1^ at kinetochores albeit to a lesser level, but over time, the localization of these proteins is lost in the large-budded cells. This suggests that the loss of Bub1 or Aurora B^Ipl1^ from kinetochores leads to failure in SAC maintenance in *sgo1Δ* cells during MT depolymerizing conditions. We hypothesize that Sgo1 regulates the SAC function through Aurora B^Ipl1^ in two ways that may not necessarily be mutually exclusive: a) by maintaining high Aurora B^Ipl1^ activity at kinetochores, thereby preventing the action of PP1 in terminating SAC signals, and b) by regulating Mps1 and Bub3/Bub1 [18,56,57] at kinetochores, thereby modulating the SAC response. The fact that we observe an increase in the levels of PP1 at kinetochores in the reduced levels of Aurora B^Ipl1^ indicates that Sgo1, more likely, acts through Aurora B^Ipl1^ to antagonize the activity of PP1 at kinetochores thereby preventing the premature SAC termination.

The fact that Sgo1 is not required for the SAC activation but for the maintenance of the SAC response via maintaining a balance between Aurora B^Ipl1^-PP1 at kinetochores also explains why *sgo1Δ mad2Δ* double mutants are synthetic lethal in the presence of MT depolymerizing drugs. The observed synergy indicates that both Sgo1 and Mad2 act in different pathways, where Sgo1 may play other roles such as biorientation of chromosomes that function (i) via the Aurora B^Ipl1^-PP1-Bub1 axis; (ii) by recruiting condensin [58,59] to the centromeres which biases sister kinetochores to bi-orient by affecting the kinetochore geometry; (iii) by maintaining the centromeric cohesion to prevent premature separation of sister chromatids, see review [40].

The mechanism of recruitment of shugoshin to kinetochores is a stratified process as it involves several proteins. Among these, Bub1 plays a critical role in targeting shugoshin to kinetochores in *S*. *cerevisiae*, *S*. *pombe*, *Xenopus laevis*, and humans [26,38,60] (Fig 6A). In humans, it has been reported that Cdk1-mediated phosphorylation of shugoshin promotes its interaction with cohesin and targeting to inner centromeres [38]. Similarly, in *S*. *cerevisiae* [61] and *S*. *pombe* [41] cohesins are known to interact with shugoshin and this association in *S*. *cerevisiae* promotes the recruitment of shugoshin to pericentromeric regions in mitosis [58,61]. Apart from cohesins, it has been shown that a heterochromatin protein, HP1 promotes centromeric recruitment of shugoshin in *S*. *pombe* [62]. It has also been reported that shugoshin and the CPC complex mutually influence each other’s localization at centromeres. In *Drosophila*, the phosphorylation of MEI-S332 (shugoshin) by Aurora B^Ipl1^ promotes its centromere localization [63,64]. Studies have also implicated the role of CENP-A and H3 nucleosomes in targeting shugoshin to centromeres as well [65–69]. Intriguingly, in *C*. *neoformans*, the checkpoint maintenance function of Sgo1 is not mediated through the Bub1-pH2A axis, as disruption of this pathway did not abolish the Sgo1 localization or the SAC maintenance function. This suggests that Bub1 is not a major player in recruiting CnSgo1 unlike the other species studied. We speculate that the Bub1 independent recruitment of CnSgo1 could be due to the non-canonical localization of the major pool of shugoshin at SPBs and along the mitotic spindle.

Altogether, this study illustrates the functional diversity of an evolutionarily conserved protein, shugoshin. A spatio-temporal balance of kinase and phosphatase activity is crucial for proper kinetochore-MT attachments and SAC signaling. Sgo1 monitors the kinetochore-MT attachment state in mitosis by balancing Aurora B^Ipl1^ and PP1 levels at kinetochores. Based on the ChIP data, it is possible that there could be a transient and/or a minor pool of shugoshin associated with the kinetochores or kinetochore proximal regions. SPBs and spindle microtubules, along with other proteins such as HP1, CPC, CENP-A, and histone H3, might promote the kinetochore proximal association of Sgo1 in *C*. *neoformans*. This kinetochore proximal localization of shugoshin may not primarily rely on Bub1 kinase function. It is also possible that CPC or the activity of polo-like kinase (Cdc5/PLK1) as reported in humans [70] may also play a role in targeting Sgo1 to SPBs. Further investigation is needed to confirm the importance of SPBs/microtubules in regulating the function of Sgo1 in this organism of high medical importance.

## Materials and methods

### Strains and media growth conditions

*C*. *neoformans* cultures were grown and maintained on YPD (1% yeast extract, 2% peptone, and 2% dextrose) and YPG (1% yeast extract, 2% peptone, and 2% galactose) media at 30°C unless specified. YPD+1M sorbitol or YPG+1M sorbitol plates were used for transformation. For the selection of transformants, 100 μg/mL of nourseothricin (NAT, clonNAT, Werner BioAgents), 180 μg/mL neomycin (G-418, Sigma-Aldrich), and 220 μg/mL of hygromycin (HiMedia) were used.

### Biolistic transformation

The biolistic transformation was done based on a previously described method [71]. Approximately 5 mL of *C*. *neoformans* culture was grown overnight, centrifuged at 4,000 rpm for 5 min, and resuspended in 300 μL sterile dH_2_O. Around 300 μL of cell suspension was spread on the YPD+1M sorbitol plate and dried. Gold micro-carrier beads (0.6 μm) coated with 2–5 μg of DNA were prepared, and 10 μL of 2.5 M CaCl_2_, 2 μL of 1M spermidine free base (Sigma-Aldrich) were added. The mixture was vortexed for 30 s, incubated for 10 min at room temperature, centrifuged, washed with 500 μL of 100% ethanol, and resuspended in 10 μL of 100% ethanol. The solution was placed on a micro-carrier disk and allowed to dry. Dried disks were placed in the biolistic transformation apparatus (Biolistic® PDS-1000/He Particle Delivery System). Helium gas pressure of approximately 1300 psi under a vacuum of a minimum of 25 inches of Hg was generated to bombard the cells with micro-carrier beads. The cells were incubated for 5–6 h in YPD+1M sorbitol medium at 30°C. Cells were then plated on YPD+NAT/NEO/HYG selection plate and incubated at 30°C for 3–4 days.

### Strains and plasmids

Lists of strains, plasmids, and primers used in this study are provided in S1–S3 Tables. The construction of *C*. *neoformans* strains and plasmids is detailed below.

### *GFP-SGO1* strains

The fluorescent fusion protein of shugoshin was generated by cloning the 1200 bp homology region and the *SGO1* promoter into the pVY7 plasmid containing the GFP and NAT sequences. GFP was tagged to the N-terminal region of the *SGO1* gene. The primers used for generating the clone are listed in S3 Table. The 1200 bp homology region and a 500 bp *SGO1* promoter region were amplified from the *SGO1* gene using *C*. *neoformans* (H99) genomic DNA. The individual PCR fragments were gel purified. The amplified regions were cloned using BamHI, SacI, and NcoI sites. The final clone was linearized using HindIII and transformed into H99 and CNVY101. The transformants obtained were confirmed by PCR.

### *sgo1Δ* strains

An overlap PCR strategy was used to delete *SGO1* gene in the desired strains. Around 1000 bp region upstream to the start codon (ATG) and 1000 bp downstream region of the stop codon of the *SGO1* ORF was amplified using *C*. *neoformans* (H99) genomic DNA. The *NEO* resistance marker fragment was amplified from the pLK25 plasmid (S2 Table). The individual PCR fragments were gel purified. Using these individual PCR amplified fragments, a final overlap cassette of ~3800 bp was constructed using primers (S3 Table). The final cassette was used to transform *C*. *neoformans*. The transformants obtained were confirmed by PCR. To delete the *SGO1* gene in different strain backgrounds, the *NEO* marker was swapped with either *NAT* or *HygB* genes amplified from the pVY7 and pSH7G plasmids (S2 Table) using the same set of primers. The *sgo1Δ* cassette was used to transform H99, CNVY108, SHR741, SHR830, IL102, IL143, CNKB003 and CNSD190 strains. The transformants obtained were screened by PCR.

### *TUB4-mCherry* strain

An overlap PCR strategy was used to tag Tub4 with mCherry at C-terminus. Primers used for overlap cassette construction are listed in S3 Table. Around 1000 bp region upstream to the stop codon (without including the stop codon) and 1000 bp 3’ UTR region after the stop codon was amplified from the *TUB4* ORF using *C*. *neoformans* (H99) genomic DNA. The mCherry sequence with antibiotic resistance marker NEO was amplified from the pLK25 plasmid (S2 Table). The individual PCR fragments were gel purified. A final overlap cassette of ~4500 bp was constructed using individual PCR fragments. The final cassette was transformed into CNSD127 strain. The transformants obtained were confirmed by PCR.

### *GAL7-3xFLAG* tagged *SCC1*, *AURORA B*^*IPL1*^, *CDC20* strains

To place the desired gene under regulatable *GAL7p* along with an N-terminal 3xFLAG sequence, an overlap PCR strategy was used. Around 1000 bp regions upstream (US) and downstream (DS) of the start codon (ATG) of the ORFs of interest were amplified using *C*. *neoformans* (H99) genomic DNA. *GAL7* promoter with *HygB* resistance gene was amplified from pSH7G plasmid, and the 3xFLAG sequence was introduced using a primer (S3 Table). The individual PCR fragments were gel purified. A final overlap cassette was assembled using the individual purified fragments. The cassettes generated were transformed into *C*. *neoformans* and the positive transformants were confirmed by PCR. The 3xFLAG tagged conditional mutants of *SCC1*, *AURORA B*^*IPL1*^, and *CDC20* generated using the strategy mentioned above are listed in S1 Table.

### *AURORA B*^*IPL1*^*-3xGFP mCherry-CENP-A* strain

The H99 strain expressing Aurk B^Ipl1^-3xGFP and mCherry-CENP-A was generated by the random integration of the plasmid pLKB71 (S2 Table). The mCherry-CENP-A construct is expressed using the *H3* promoter. To create the strain, the plasmid backbone of pLKB71 was digested with HindIII and transformed into CNNV113, and the transformants were selected on the YPD-agar plate containing hygromycin selection. The colonies obtained were screened using a Zeiss Axio Observer 7 widefield microscope for the transformants positive for the mCherry-CENP-A signal.

### *GFP-PP1 mCherry-CENP-A* strain

The N-terminal GFP fusion protein of PP1 was generated by cloning around 1000 bp homology region and PP1 promoter into the pVY7 plasmid containing GFP and NAT sequences. Around 955 bp PP1 promoter region and 1000 bp PP1 homology region were amplified from PP1 ORF using *C*. *neoformans* (H99) genomic DNA. The PCR amplicons were gel purified. The digested amplicons of promoter and homology region were cloned into pVY7 using SacI, NcoI, and SpeI sites, respectively. The final clone was linearized using BglII and transformed into the CNVY101 strain, and the positive transformants were confirmed by PCR.

### *GFP-sgo1-K382A-SH1*, *GFP-SGO1-SH1* and *GFP-sgo1-K382A-SH1 mCherry-CENP-A* strains

The N-terminal GFP fusion protein of wild-type and mutant (K382A) allele of *SGO1* was generated by cloning *SGO1* promoter, *SGO1* ORF, and 3’UTR into the pSD29 plasmid (S2 Table) containing the GFP, HygB and *SAFE HAVEN* sequences. Around 2340 bp of *SGO1* ORF 315 bp of *SGO1* promoter, and 436 bp of 3’UTR were amplified from the *SGO1* gene using *C*. *neoformans* (H99) genomic DNA. The K382A mutation in the *SGO1* ORF was introduced using overlap PCR. The primers used are listed in S3 Table. The individual PCR fragments were gel purified. The purified fragments of the *SGO1* promoter, *SGO1* ORF, and 3’UTR were cloned sequentially using SpeI, BamHI, HpaI, NheI, and ApaI sites. The final clone was linearized using XcmI and transformed into CNSD113. The transformants obtained were confirmed by PCR, and Sanger sequencing was performed to confirm the presence of the *K382A* mutation. The strain harbouring *GFP-sgo1-K382A* allele at *SAFE HAVEN* locus was transformed with mCherry-CENP-A-NAT plasmid linearized using BglII and the positive transformants were confirmed by microscopy.

### *h2a-T121A* GFP-SGO1 mCherry-CENP-A strain

A 1503 bp fragment of H2A ORF and 3’ UTR was amplified by PCR using *C*. *neoformans* (H99) genomic DNA and the T121A mutation was introduced into the 1503 bp fragment using overlap PCR strategy. A second fragment containing a Hygromycin marker was amplified from pSH7G. The homology region downstream of H2A 3’ UTR was amplified by PCR using *C*. *neoformans* (H99) genomic DNA. The primers used for generating the construct are listed in S3 Table. The three PCR fragments were subsequently fused by overlap PCR. The final overlap product was used to transform the CNSD125 strain using the biolistic transformation method, and the transformants obtained were confirmed by PCR, and Sanger sequencing was performed to confirm the presence of mutation within the gene.

### *bub1-kd 3xFLAG GFP-SGO1 mCherry-CENP-A* strain

The 3xFLAG-tagged bub1-kd cassette was generated by cloning the 1200 bp homology region of BUB1 ORF harboring point mutations into the pSH7G plasmid containing the HYG sequences. The primers used for generating the clone are listed in S3 Table. The 1200 bp homology region was amplified from the *bub1-kd* allele using genomic DNA obtained from the IL143 (*GFP-bub1-kd*) strain. The individual PCR fragments were gel purified. The amplified regions were cloned using SpeI and KpnI sites. The final clone was linearized using ApaI and transformed into CNSD125 background using the biolistic transformation method. The transformants obtained were confirmed by PCR.

### Thiabendazole sensitivity assay

For assaying sensitivity to thiabendazole, the O.D_600_ values of overnight cultures of wild-type and mutants used in this study were estimated, and serial dilutions starting from 10^5^ cells to 10^1^ cells were spotted on YPD-agar, DMF (Dimethyl Formamide); control, and YPD-agar plates with different concentrations of thiabendazole (2.0, 2.5, 3.0 and 4.0 μg/mL). The plates were incubated at 30°C for 24 to 48 h and imaged using a Bio-Rad chemidoc imaging system.

### Synchronization of *C*. *neoformans* by inducing physical hypoxia

For the synchronization of *C*. *neoformans* cells, we have used a protocol developed by Ohkusu et al., [42] which utilizes a two-step protocol for the synchronization. *C*. *neoformans* cells, when grown in hypoxia conditions, result in unbudded G2 arrest, and cells release synchronously once shifted to extensive aeration conditions. Hypoxia synchrony is a two-step process. First, cells were grown at moderate aeration to 4.0 O.D_600_/mL in a 250 mL flask (Borosil Erlenmeyer flask with a screw cap was used for this experiment) with 50 mL of 1% YPD at 100 rpm, 30°C. In the second step, the culture was diluted 1.5 times with fresh 1% YPD (final volume of 125 mL) and then incubated further for 5 h. The second step induces physical hypoxia, and at the end of the incubation, most cells get arrested at the unbudded G2 stage.

For the synchronization experiments, we used an overnight culture grown in 1% YPD to inoculate the secondary culture at 0.1 O.D_600_/mL in 1% YPD and grown to 1 O.D_600_/mL at 180 rpm, 30°C. This log phase culture was used for hypoxia synchrony, as mentioned above. The percentage of cells in the unbudded G2 stage was confirmed using a microscope. The culture was diluted to 1 O.D_600_/mL using fresh media, transferred to a sterile falcon or conical flask with appropriate culture volumes, and released under extensive aeration conditions (180 rpm, 30°C).

### GFP-Bub1 and Aurora B^Ipl1^-3xGFP retention assay

To study the localization dynamics of GFP-Bub1 and Aur B^Ipl1^-3xGFP in response to nocodazole treatment, wild-type, and *sgo1Δ* mutants were subjected to hypoxia synchrony, and the cells were released into 1% YPD media containing 1 μg/mL nocodazole. Time points were collected every 40 minutes until 280 min. The cells collected were imaged using a Zeiss Axio Observer 7 widefield microscope. A circular ROI of 12*12 or 6*6 pixels was used to measure the signal intensities. The presence or absence of GFP-Bub1 signals was scored manually. The cells that show bright punctate localization of Bub1 above the cell background were considered positive for Bub1.

### Imaging GFP-Sgo1 localization in *mCherry-CENP-A* and *TUB4-mCherry* strains

For imaging GFP-Sgo1 colocalization with CENP-A- and Tub4-mCherry tagged proteins, we synchronized cells using physical hypoxia. Cells were released into 1% YPD and collected every 10 min starting from 30 to 90 min. Cells were imaged using a Zeiss Axio Observer 7 widefield microscope.

### Cell cycle synchronization using thiabendazole (TBZ)

In order to obtain metaphase cells, the overnight-grown culture of *Cryptococcus* was inoculated at 0.2 O.D_600_/mL and grown to log phase. The log phase culture was treated with 10 μg/mL thiabendazole for 2 h. The cells arrested at G2/M were washed twice with 2% YPD and released into fresh pre-warmed 2% YPD by incubating at 30°C, 180 RPM for 2 min. The cells were harvested by spinning at 5000 RPM for 30 seconds, washed twice with sterile water, and imaged using a Zeiss Axio Observer 7 widefield microscope.

### Line scan analysis of GFP-Sgo1 signal at metaphase stage

For measuring the fluorescent intensity of Sgo1 at metaphase in wild-type and the *bub1-kd*, *h2a-T121A*, and *sgo1-K382A* strains, a line of 2-pixel thickness and a length of approximately 3.4 μm was drawn using a line tool available in Fiji [72]. A single Z-plane containing both SPB and spindle signals was considered for intensity measurement. The fluorescence intensity values along the line were measured using the plot profile function. The intensity values of the GFP signal obtained from different cells were averaged and divided by the average background of the cells. The normalized intensity values were plotted using GraphPad Prism.

### Budding index calculation

The proportion of unbudded, small-budded, and large-budded cells was calculated based on the budding index (diameter of daughter cell/diameter of mother cell). The diameters of both mother and daughter cells were calculated along the mother-daughter axis using the line tool in Fiji [72].

### Fluorescence microscopy

For widefield microscopy, Zeiss Axio Observer 7 equipped with 100x Plan Apochromat 1.4 NA objective, Colibri 7 LED light source, motorized XYZ stage and PCO.edge 4.2 sCMOS camera was used. Images were acquired at 2x2 or 4x4 binning. Zen blue edition (2.3) software was used for controlling the microscope components and acquisition. For imaging, cells grown in culture media were washed twice with autoclaved dH_2_O and resuspended in water. The cell suspension was placed on microscope cover glass and imaged.

### Time-lapse microfluidic assays

The utility of microfluidics for single-cell analysis in *Cryptococcus* was demonstrated previously in studies of aging [73]. We used the Alcatras cell traps [74] incorporated into devices allowing for use with multiple strains [75,76]. Devices were moulded in polydimethylsiloxane (PDMS) from a SU8-patterned wafer with an increased thickness of 7 μm to accommodate the larger size of *C neoformans* cells compared to *S cerevisiae* (manufactured by Micro-resist, Berlin, design available on request). Imaging chambers for individual strains were isolated by arrays of PDMS pillars separated by 2 μm gaps. This prevents the intermixing of strains while cells experience identical media conditions. Before use, the devices were filled with synthetic complete (SC) media, supplemented with 0.2 g/L glucose, and containing 0.05% w/v bovine serum albumin (Sigma) to reduce cell-cell and cell-PDMS adhesion. Cells pre-grown to logarithmic phase in the same media (lacking the BSA) were injected into the device. An EZ flow system (Fluigent) delivered media at 10 μl per minute to the flow chambers and performed the switch to media containing nocodazole after 5 hours. This media also contained Cy5 dye to allow monitoring of the timing of the media switch. Image stacks were captured at 2-minute intervals at 4 stage positions for each strain, using a Nikon TiE epifluorescence microscope with a 60x oil-immersion objective (NA 1.4), a Prime95b sCMOS camera (Teledyne Photometrics) and OptoLED illumination (Cairn Research). Image stacks had 5 Z-sections, separated by 0.6 μm, captured using a piezo lens positioning motor (Pi).

### *Time-lapse* microfluidic assay image processing and analysis

Time-lapse assays were performed as described [34]. Cell outlines were segmented using the baby algorithm [77]. To quantify the kinetochore retention time of GFP-Bub1 fluorescence, we create a projection of the maximum values from all GFP sections, then divide the median fluorescence of the brightest 5 pixels within each cell by the median fluorescence of the cell as a whole. This ratio reliably measures protein aggregation at an organelle and has been extensively used for quantification [78–80]. In the absence of nocodazole, cells with any outlier values for the ratio, defined as values greater than 3 scaled median absolute deviations away from the median, are removed. Cells present for fewer than 80% of recorded time points are also removed. The ratios were normalized for each strain by dividing the mean value for wild-type cells in the absence of nocodazole, measured in the same experiment. To calculate the kinetochore retention times, a threshold of 1.36 (Fig 2F and 2G) and 1.11 (S4A and S4B Fig) was applied to the ratio values at each time point to determine the presence or absence of Bub1 at the kinetochore (Figs 2G and S4B). Small gaps in localization (due to kinetochore signal dropping briefly out of focus) were removed by applying a 1-dimensional morphological closing to the thresholded image, with a 3-pixel (3 timepoint or 6 minutes) structuring element. For the temporal heat maps (Figs 2F and S4A), the data for 30 cells present for the whole experiment are selected for inclusion from each strain pseudo-randomly using the Matlab rand function. There were a small number of outlier values within the heat maps, resulting from a fault in the LED control equipment. These were removed by replacing any values in the 5th percentile of a cell trace with the median value for all time points. The raw data and code used in data processing and plotting are available on request.

### Flow cytometry

5 mL of *C*. *neoformans* culture was grown overnight, and 2 O.D_600_ cells were taken and washed with sterile dH2O and fixed in 1 mL of 70% ethanol overnight at 4°C. Fixed cells were centrifuged at 4000 rpm and washed with 1 mL NS buffer(10 mM Tris-HCl pH 7.5, 250 mM sucrose, 1 mM EDTA (pH 8.0), 1 mM MgCl_2_, 0.1 mM CaCl_2_, and 7 mM β-mercaptoethanol) and resuspended in 200 μL of NS buffer added with 2 μg of RNase and incubated at 37°C for 3–4 h. Propidium Iodide (final concentration 12 μg/mL) was added to this mixture and incubated for 30 min at room temperature in the dark. 50 μL of the sample was suspended in 2 mL of 1x PBS (Phosphate buffered saline) solution, vortexed, and sonicated for 10 s at 30% Amp. 30,000 cells were analyzed by flow cytometry using FL2-A channel on (FACSAria III; BD Biosciences) at a rate of 500–2,000 events/s.

### Western blotting

For immunoblotting, whole cell lysates were prepared by harvesting 10 O.D_600_ cells cultured using YPD. The cells were washed twice with sterile water and resuspended in 15% TCA (Trichloroacetic acid), and samples were frozen in a -20°C freezer. The cell suspension was thawed on ice and lysed using a mini-bead beater (Biospec product) with 1 min ON and 5 min OFF for three cycles at 3000 RPM. The cell lysate was centrifuged at 13000 RPM at 4°C for 10 min, and the supernatant was discarded. The pellet was resuspended in 2x sample buffer (0.1 M Tris HCl pH 6.8, 20% glycerol, 4% SDS, 715 mM β-mercaptoethanol and 0.2% bromophenol blue) and incubated for 5 min in a boiling water bath. The samples were cleared by spinning at 13000 RPM for 3 min, and the supernatant was loaded immediately onto an SDS-PAGE gel and separated. The proteins were transferred to a PVDF membrane (Bio-Rad) using the wet transfer method with a transfer time of two and a half hours at 90 V. The transfer efficiency was estimated by staining the blot with Ponceau S solution. The membrane was blocked using 3% skimmed milk and 2% BSA dissolved 1x PBST (0.1% Tween-20) solution at room temperature for 60 minutes. Post blocking, the blot was incubated in 2% skimmed milk and 1% BSA dissolved in 1x PBST solution containing anti-GFP at 1:3000 dilution overnight at 4°C. The membrane was washed thrice for 10 min with 1x PBS solution containing 0.1% tween-20. The blot was incubated with an anti-mouse secondary antibody (1:10000 dilution in 2% skimmed milk and 1% BSA dissolved in 1x PBST solution) for 1 hour at room temperature. The membrane was washed thrice for 10 min with 1x PBS + 0.1% tween-20, and the blot was rinsed in 1x PBS and was developed using Bio-Rad clarity ECL solution and imaged using Bio-Rad Chemidoc imaging system.

### Chromatin Immunoprecipitation (ChIP)

The ChIP protocol was adapted from [81,82]. ChIP was performed in metaphase-arrested cells by depleting Scc1. Overnight cultures of CNSD185 strain grown in 2% YPG (permissive media) were washed twice with 2% YPD and inoculated at 0.2 O.D_600_/mL in 150 mL of 2% YPD (Non-permissive) media. The cells were grown for 6 h at 30°C, 180 RPM. Post 6 h incubation, the extent of metaphase arrest was assessed using microscopy, and the culture was fixed by adding freshly prepared methanol-free formaldehyde at 3% final concentration and incubated at 25°C, 100 RPM for 35 minutes. The fixed cells were quenched with 1.5 M Tris HCl pH 8.0 for 5 mins at 25°C, 100 RPM. The cells were pelleted and washed with ice-cold water flash frozen using liquid Nitrogen, and stored at -80°C. The cells were lysed in a buffer containing 50 mM HEPES pH 7.4, 1% Triton-X, 140 mM NaCl, 0.1% Na-deoxycholate, 1 mM EDTA, 1x Protease Inhibitor Cocktail (Sigma) with 10 cycles of bead beating using a mini-beadbeater (BioSpec product) at 3500 RPM for 2 mins ON and 5 mins OFF on ice.

The lysate was transferred to a chilled 15 mL falcon on ice, and the cross-linked chromatin was fragmented using an ultrasonicator (Diagenode Bioruptor Pico) at high frequency, 30 s ON, 30 s OFF mode for 50 cycles to achieve the desired 250–500 size range. The sample was transferred to a chilled 2 mL tube, and centrifuged at 10000 RPM for 10 min at 4°C. The debris-free supernatant was carefully transferred to a fresh 2 mL tube and incubated with 20 μL of control trap beads (Chromo Tek) at 11 RPM for 3 h at 4°C. After incubation, bead-free supernatant was recovered by centrifugation at 500 RCF for 2 min, and GFP-trap and block beads were added to the respective tubes. The tubes were incubated at 11 RPM rotation for 12 h at 4°C. After incubation, the beads were recovered by centrifugation at 1000 RCF for 1 min at RT, and extensive washes were performed at RT with intermittent agitation at 11 RPM for 5 min. First, the beads were washed with 1 mL lysis buffer (50 mM HEPES pH 7.5, 140 mM NaCl, 1% Triton-X 100, 0.1% sodium deoxycholate, 2 mM PMSF), recovered by centrifugation at 1000 RCF for 1 min at RT and then washed 2 times each with the following buffers, with recovery at 1000 RCF for 1 min following each wash: (i) 20 mM Tris HCl pH 8.0, 200 mM NaCl, 2 mM EDTA, 1% Triton-X-100, 0.1% SDS. (ii) 20 mM Tris HCl pH 8.0, 500 mM NaCl, 2 mM EDTA, 1% Triton-X-100, 0.1% SDS. (iii) 20 mM Tris HCl pH 8.0, 250 mM LiCl, 2 mM EDTA, 1% NP-40, 1% sodium-deoxycholate, pH 8.0. (iv) TE: 10 mM Tris HCl pH 8.0, 1 mM EDTA. The IP complex was recovered by incubating the beads with 250 μL of elution buffer (10 mM Tris HCl pH 8.0, 1 mM EDTA, 1% SDS, 200 mM NaCl) at 65°C for 15 min with intermittent flicking. The process was repeated with another 250 μL buffer and pooled to obtain a 500 μL IP sample. The input volume was adjusted to 500 μL with an elution buffer. For de-crosslinking, 20 μL of 5 N NaCl was added to each sample and incubated overnight at 65°C. Next day, 20 μL 1M Tris-HCl pH 6.8, and 10 μL 0.5 M EDTA pH 8.0 were added to each tube and treated with Proteinase K (2 μL/sample) at 42°C for 1 h followed by RNase treatment (5 μL of 10 mg/mL RNase A/sample) at 37°C for 1 h. The DNA was extracted with an equal volume of Tris pH 8.0-saturated phenol:chloroform:isoamyl alcohol (25:24:1) and precipitated (with 1 mL of 100% ethanol with 100 μL 3 M sodium acetate pH 5.3) overnight at -80°C. The DNA was pelleted by centrifugation at 14000 RPM for 30 min at 4°C, washed with chilled 70% ethanol, air-dried, and finally resuspended in 20 μL TE pH 8.0. The ChIP-qPCR reactions were carried out in a total 10 μL volume using CEN and Non-CEN primers listed in S3 Table.

### Statistics and microscope image analysis

P-values were assessed by unpaired, two-tailed t-test or two-way ANOVA with Tukey’s multiple comparison test using GraphPad Prism 9.00 (GraphPad software). Error bars represent the standard deviation (SD) or standard error of the mean (SEM) as mentioned for each experiment. N for each experiment is mentioned in the figure legends. Fiji software [72] was used to process and analyze microscope images. Fluorescence intensity values of signals were quantified by drawing a circular region of interest (ROI) of defined pixel size on the sum intensity Z-projected images (Figs 2E, 3C and 3G) or on single Z-plane images (Fig 4C and 4E). The intensity values were calculated by subtracting the product of mean background fluorescence intensity and area of the ROI from integrated density, and the resultant values were plotted using GraphPad Prism.

## Supporting information

S1 Fig *C*. *neoformans* Sgo1, carrying a conserved coiled-coil domain and the SGO motif, is required to maintain SAC arrest in nocodazole-treated cells. **(A)** The protein sequence of *C*. *neoformans* Sgo1. **(B)** Multiple sequence alignment (MSA) of the CnSgo1 N-terminal coiled-coil and the C-terminal SGO motif with vertebrate and invertebrate species (Cn- *C*. *neoformans*; Sc- *Saccharomyces cerevisiae*; Sp- *Schizosaccharomyces pombe*; Hs- *Homo sapiens*; Mm- *Mus musculus*; Xl- *Xenopus laevis*; Dm- *Drosophila melanogaster*). Multiple sequence alignment of Sgo1 homologs was performed using Clustal Omega [83], and the alignment was formatted using Jalview 2 [84]. *Represent amino acid residues involved in PP2A binding. The dark blue circle represents the key residue (K382 in *C*. *neoformans*) required for Bub1-mediated kinetochore proximal centromere localization of shugoshin. The teal blue colored box represents the coiled-coil domain. The light blue colored box represents the conserved basic SGO motif. (C) Microscopic images of GFP-H4 in CNVY108 (*SGO1 MAD2*), CNSD117 (*sgo1Δ*), SHR866 (*mad2Δ*), CNSD148 (*sgo1Δ mad2Δ*) treated with nocodazole (1 μg/mL) for 120- and 240-min. Scale bar, 5 μm. (D) Bar graphs representing the proportion of unbudded, small-budded, large-budded, and tri-budded cells. N = 3, n>100 cells counted in each experiment. (E) Bar graphs representing the proportion of large-budded cells of indicated phenotypes, N = 3, n>100 cells counted in each experiment.
(TIF)

S2 Fig *sgo1Δ bgi1Δ* double mutant cells exhibit enhanced sensitivity to MT depolymerizing drug. A ten**-**fold serial dilution spotting assay to score for the sensitivity of the wild-type CNVY108 (*SGO1 BGI1 GFP-H4*), CNSD117 (*sgo1Δ GFP-H4)*, SHR741 (*mad2Δ GFP-H4*), SHR830 (*bgi1Δ GFP-H4*), CNSD148 (*sgo1Δ mad2Δ GFP-H4*) and CNSD163 (*sgo1Δ bgi1Δ GFP-H4*) to thiabendazole. No drug represents DMF (Dimethyl formamide) only. The plates were incubated at 30°C for 24 h.
(TIF)

S3 Fig The proportion of large-budded cells scored for the presence or absence of GFP-Bub1 signal post nocodazole treatment. Bar graphs representing the proportion of large-budded cells obtained post nocodazole treatment in the indicated strains, N = 3, n>100, cells counted in each experiment. Errors bars represent SD.
(TIF)

S4 Fig *bub1-kd sgo1Δ* double mutant cells behave similar to single *sgo1Δ* mutant cells. **(A)** Microfluidics assay to determine the retention time of GFP-Bub1 at kinetochores in response to nocodazole treatment. Temporal heat maps of 30 randomly selected cells are shown. The heat map represents the changes in the kinetochore localization signal of GFP-Bub1 over time. Each bright track on the *y*-axis of the heat map represents GFP-Bub1 signals from an individual cell (median of the brightest 5 pixels in each cell divided by the overall cell median brightness). The length of each bright track along the *x*-axis represents the time (min). The time of addition of nocodazole (2.5 μg/mL) is considered as 0 hr. Images are taken every 2 min for 10 h. Assay was performed using IL102 (*GFP-BUB1*), IL143 (*GFP-bub1-kd*), CNSD176 (*GFP-BUB1 sgo1Δ*), and CNSD173 (*GFP-bub1-kd sgo1Δ*) strains. **(B)** Bar graphs representing the quantitative analysis of GFP-Bub1 retention time at kinetochores obtained from microfluidics assays in the above-indicated strains. n = 24, error bars represent SEM.
(TIF)

S5 Fig Aurora B^Ipl1^ localizes to the kinetochore (CENP-A) during G2 and mitosis and exhibits spindle-like localization. **(A)** Microscopic images of Aurora B^Ipl1^-3xGFP localized with a kinetochore protein mCherry-CENP-A at G2, metaphase, and anaphase stages of the cell cycle. **(B)** Colocalization of Aurora B^Ipl1^-3xGFP with mCherry-CENP-A when treated with nocodazole (1 μg/mL) for 120 min. Scale bar, 5 μm.
(TIF)

S6 Fig The proportion of large-budded cells scored for the presence or absence of Aurora B^Ipl1^-3xGFP signal post nocodazole treatment. Bar graphs representing the proportion of large-budded cells obtained post nocodazole treatment in the indicated strains, N = 3, n>100, cells counted in each experiment. Errors bars represent SD.
(TIF)

S7 Fig Aurora B^Ipl1^ mutants fail to arrest at metaphase in response to nocodazole treatment. **(A)** Microscopic images of CNNV104 (*GFP-H4 GAL7-AURORA B*^*IPL1*^) grown in permissive and non-permissive media in the presence and absence of nocodazole. Scale bar, 5 μm. Representative images of cells treated with nocodazole and depleted of Aurora B^Ipl1^ for 240 and 480 min were shown. **(B)** *Left*, bar graphs representing the proportion of unbudded, small-budded, large-budded, and tri-budded cells. *Right*, bar graphs representing the proportion of large-budded cells of indicated phenotype, N = 2, n>100 cells counted for each experiment.
(TIF)

S8 Fig Sequence conservation of *C*. *neoformans* PP1. Multiple sequence alignment of PP1 homolog of *C*. *neoformans* (CNAG_03706) with vertebrate and invertebrate species (Cn- *C*. *neoformans*; Sc- *S*. *cerevisiae*; Sp- *S*. *pombe*; Ca- *Candida albicans*; Hs- *H*. *sapiens*; Mm- *M*. *musculus*; Dm- *D*. *melanogaster*) was performed using Clustal Omega [83] and the alignment was formatted using Jalview 2 [84]. Highly conserved regions are shaded in dark blue.
(TIF)

S9 Fig Shugoshin responds to tensionless kinetochore-MT attachments. **(A)** Pairwise sequence alignment of Scc1 homologs of *C*. *neoformans* (CNAG_01023) and *S*. *cerevisiae* by Clustal Omega [83], and formatted using Jalview 2 [84]. Highly conserved regions are shaded in dark blue. The two domains corresponding to N- and C-terminal Scc1 domains (E-values of 2.0e-31 and 2.2e-15, respectively) of CnScc1 are highlighted in violet and green boxes. **(B)** Schematic of the domains present in *C*. *neoformans* Scc1 protein. **(C)** Plate photographs of strains expressing Scc1 under the regulatable *GAL7* promoter grown in media containing glucose (non-permissive) and galactose (permissive). **(D)** Schematic to check the response of Scc1 repression in *SGO1* and *sgo1Δ* backgrounds. **(E)** Microscopic images of GFP-H4 tagged CNSD181 (*GAL7-3xFLAG-SCC1*) and CNSD182 (*sgo1Δ GAL7-3xFLAG-SCC1*) strains depleted of Scc1. Representative images of cells depleted of Scc1 for 4 and 6 h were shown. Scale bar, 5 μm. **(F)** Bar graphs representing the percentage of unbudded, small-budded, large-budded, and tri-budded cells scored. N = 2, n>100 cells for each experiment, error bars represent SD.
(TIF)

S10 Fig Conservation of histone H2A sequence in *C*. *neoformans* and expression levels of GFP-Sgo1 in various mutant strains. **(A)** Multiple sequence alignment of H2A homologs obtained from *C*. *neoformans* (Cn), *S*. *cerevisiae* (Sc), *S*. *pombe* (Sp) and *H*. *sapiens* (Hs). The alignment was performed using Clustal Omega [83] and formatted using Jalview 2 [84]. The dark blue shaded regions represent highly conserved amino acid residues. Arrow indicates the conserved T121/S120 residue phosphorylated by Bub1. **(B)** Western blot showing the expression levels of GFP-Sgo1 at metaphase in the indicated strains. * Indicates non-specific band used as a loading control. Arrows indicate two bands of GFP-Sgo1. *sgo1Δ* lane represents no tag control.
(TIF)

S11 Fig Schematic highlighting the sub-cellular localization of shugoshin reported in various species. *Left*, schematic highlighting the spatial positioning of SPB/centrosome and centromeres in yeasts and metazoans. *Right*, localization of shugoshin with respect to the kinetochore, MTOCs (centrosomes or SPBs), spindle MTs, and telomeres. Filled and open squares represent the presence or absence of shugoshin at the indicated sub-cellular locations. The data was compiled from reviews [39, 40]. ^[^^1^^,^^2^^]^ [70, 85], ^[3*]^ [86], this study has shown that the purified N-terminal region of shugoshin is capable of binding to MTs *in* vitro. ^[^^4^^]^ [25] and ^[^^5^^]^ [87].
(TIF)

S1 Table List of strains. (DOCX)

S2 Table List of plasmids. (DOCX)

S3 Table List of primers. (DOCX)

## References

[1] Musacchio A, Desai A. A Molecular View of Kinetochore Assembly and Function. Biology. 2017 Jan 24;6(1). doi: 10.3390/biology6010005 28125021 PMC5371998

[2] Lara-Gonzalez P, Pines J, Desai A. Spindle assembly checkpoint activation and silencing at kinetochores. Semin Cell Dev Biol. 2021 Sep;117:86–98. doi: 10.1016/j.semcdb.2021.06.009 34210579 PMC8406419

[3] Zich J, Hardwick KG. Getting down to the phosphorylated ’nuts and bolts’ of spindle checkpoint signalling. Trends Biochem Sci. 2010 Jan;35(1):18–27. doi: 10.1016/j.tibs.2009.09.002 19836959

[4] Musacchio A, Salmon ED. The spindle-assembly checkpoint in space and time. Nat Rev Mol Cell Biol. 2007 May;8(5):379–93. doi: 10.1038/nrm2163 17426725

[5] Hardwick KG, Johnston RC, Smith DL, Murray AW. MAD3 encodes a novel component of the spindle checkpoint which interacts with Bub3p, Cdc20p, and Mad2p. The Journal of cell biology. 2000 Mar 6;148(5):871–82. doi: 10.1083/jcb.148.5.871 10704439 PMC2174553

[6] Musacchio A. The Molecular Biology of Spindle Assembly Checkpoint Signaling Dynamics. Current biology: CB. 2015 Oct 19;25(20):R1002–18. doi: 10.1016/j.cub.2015.08.051 26485365

[7] Sudakin V, Chan GK, Yen TJ. Checkpoint inhibition of the APC/C in HeLa cells is mediated by a complex of BUBR1, BUB3, CDC20, and MAD2. The Journal of cell biology. 2001 Sep 3;154(5):925–36. doi: 10.1083/jcb.200102093 11535616 PMC2196190

[8] Cohen-Fix O, Peters JM, Kirschner MW, Koshland D. Anaphase initiation in Saccharomyces cerevisiae is controlled by the APC-dependent degradation of the anaphase inhibitor Pds1p. Genes Dev. 1996 Dec 15;10(24):3081–93. doi: 10.1101/gad.10.24.3081 8985178

[9] King RW, Deshaies RJ, Peters JM, Kirschner MW. How proteolysis drives the cell cycle. Science. 1996 Dec 6;274(5293):1652–9. doi: 10.1126/science.274.5293.1652 8939846

[10] Biggins S, Murray AW. The budding yeast protein kinase Ipl1/Aurora allows the absence of tension to activate the spindle checkpoint. Genes Dev. 2001 Dec 1;15(23):3118–29. doi: 10.1101/gad.934801 11731476 PMC312839

[11] Tanaka TU, Rachidi N, Janke C, Pereira G, Galova M, Schiebel E, et al. Evidence that the Ipl1-Sli15 (Aurora kinase-INCENP) complex promotes chromosome bi-orientation by altering kinetochore-spindle pole connections. Cell. 2002 Feb 8;108(3):317–29. doi: 10.1016/s0092-8674(02)00633-5 11853667

[12] Hauf S, Cole RW, LaTerra S, Zimmer C, Schnapp G, Walter R, et al. The small molecule Hesperadin reveals a role for Aurora B in correcting kinetochore-microtubule attachment and in maintaining the spindle assembly checkpoint. The Journal of cell biology. 2003 Apr 28;161(2):281–94. doi: 10.1083/jcb.200208092 12707311 PMC2172906

[13] Pinsky BA, Kung C, Shokat KM, Biggins S. The Ipl1-Aurora protein kinase activates the spindle checkpoint by creating unattached kinetochores. Nat Cell Biol. 2006 Jan;8(1):78–83. doi: 10.1038/ncb1341 16327780

[14] Cheeseman IM, Chappie JS, Wilson-Kubalek EM, Desai A. The conserved KMN network constitutes the core microtubule-binding site of the kinetochore. Cell. 2006 Dec 1;127(5):983–97. doi: 10.1016/j.cell.2006.09.039 17129783

[15] Ciferri C, Pasqualato S, Screpanti E, Varetti G, Santaguida S, Dos Reis G, et al. Implications for kinetochore-microtubule attachment from the structure of an engineered Ndc80 complex. Cell. 2008 May 2;133(3):427–39. doi: 10.1016/j.cell.2008.03.020 18455984 PMC4754795

[16] Kallio MJ, McCleland ML, Stukenberg PT, Gorbsky GJ. Inhibition of aurora B kinase blocks chromosome segregation, overrides the spindle checkpoint, and perturbs microtubule dynamics in mitosis. Current biology: CB. 2002 Jun 4;12(11):900–5. doi: 10.1016/s0960-9822(02)00887-4 12062053

[17] Petersen J, Hagan IM. S. pombe aurora kinase/survivin is required for chromosome condensation and the spindle checkpoint attachment response. Current biology: CB. 2003 Apr 1;13(7):590–7. doi: 10.1016/s0960-9822(03)00205-7 12676091

[18] Saurin AT, van der Waal MS, Medema RH, Lens SM, Kops GJ. Aurora B potentiates Mps1 activation to ensure rapid checkpoint establishment at the onset of mitosis. Nat Commun. 2011;2:316. doi: 10.1038/ncomms1319 21587233 PMC3113227

[19] Santaguida S, Vernieri C, Villa F, Ciliberto A, Musacchio A. Evidence that Aurora B is implicated in spindle checkpoint signalling independently of error correction. EMBO J. 2011 Apr 20;30(8):1508–19. doi: 10.1038/emboj.2011.70 21407176 PMC3102279

[20] Pinsky BA, Nelson CR, Biggins S. Protein phosphatase 1 regulates exit from the spindle checkpoint in budding yeast. Current biology: CB. 2009 Jul 28;19(14):1182–7. doi: 10.1016/j.cub.2009.06.043 19592248 PMC2731492

[21] Liu D, Vleugel M, Backer CB, Hori T, Fukagawa T, Cheeseman IM, et al. Regulated targeting of protein phosphatase 1 to the outer kinetochore by KNL1 opposes Aurora B kinase. The Journal of cell biology. 2010 Mar 22;188(6):809–20. doi: 10.1083/jcb.201001006 20231380 PMC2845083

[22] Roy B, Verma V, Sim J, Fontan A, Joglekar AP. Delineating the contribution of Spc105-bound PP1 to spindle checkpoint silencing and kinetochore microtubule attachment regulation. The Journal of cell biology. 2019 Dec 2;218(12):3926–42. doi: 10.1083/jcb.201810172 31649151 PMC6891095

[23] Indjeian VB, Stern BM, Murray AW. The centromeric protein Sgo1 is required to sense lack of tension on mitotic chromosomes. Science. 2005 Jan 7;307(5706):130–3. doi: 10.1126/science.1101366 15637284

[24] Vanoosthuyse V, Prykhozhij S, Hardwick KG. Shugoshin 2 regulates localization of the chromosomal passenger proteins in fission yeast mitosis. Mol Biol Cell. 2007 May;18(5):1657–69. doi: 10.1091/mbc.e06-10-0890 17301288 PMC1855032

[25] Sane A, Sridhar S, Sanyal K, Ghosh SK. Shugoshin ensures maintenance of the spindle assembly checkpoint response and efficient spindle disassembly. Mol Microbiol. 2021 Oct;116(4):1079–98. doi: 10.1111/mmi.14796 34407255

[26] Kawashima SA, Yamagishi Y, Honda T, Ishiguro K, Watanabe Y. Phosphorylation of H2A by Bub1 prevents chromosomal instability through localizing shugoshin. Science. 2010 Jan 8;327(5962):172–7. doi: 10.1126/science.1180189 19965387

[27] Fernius J, Hardwick KG. Bub1 kinase targets Sgo1 to ensure efficient chromosome biorientation in budding yeast mitosis. PLoS Genet. 2007 Nov;3(11):e213. doi: 10.1371/journal.pgen.0030213 18081426 PMC2098806

[28] Heitman J. Microbial Pathogens in the Fungal Kingdom. Fungal Biol Rev. 2011 Mar 1;25(1):48–60. doi: 10.1016/j.fbr.2011.01.003 21528015 PMC3081590

[29] Kronstad JW, Attarian R, Cadieux B, Choi J, D’Souza CA, Griffiths EJ, et al. Expanding fungal pathogenesis: Cryptococcus breaks out of the opportunistic box. Nat Rev Microbiol. 2011 Mar;9(3):193–203. doi: 10.1038/nrmicro2522 21326274 PMC4698337

[30] Kozubowski L, Yadav V, Chatterjee G, Sridhar S, Yamaguchi M, Kawamoto S, et al. Ordered kinetochore assembly in the human-pathogenic basidiomycetous yeast Cryptococcus neoformans. mBio. 2013 Oct 1;4(5):e00614–13. doi: 10.1128/mBio.00614-13 24085781 PMC3791896

[31] Sutradhar S, Yadav V, Sridhar S, Sreekumar L, Bhattacharyya D, Ghosh SK, et al. A comprehensive model to predict mitotic division in budding yeasts. Mol Biol Cell. 2015 Nov 5;26(22):3954–65. doi: 10.1091/mbc.E15-04-0236 26310442 PMC4710229

[32] van Hooff JJ, Tromer E, van Wijk LM, Snel B, Kops GJ. Evolutionary dynamics of the kinetochore network in eukaryotes as revealed by comparative genomics. EMBO Rep. 2017 Sep;18(9):1559–71. doi: 10.15252/embr.201744102 28642229 PMC5579357

[33] Sridhar S, Hori T, Nakagawa R, Fukagawa T, Sanyal K. Bridgin connects the outer kinetochore to centromeric chromatin. Nat Commun. 2021 Jan 8;12(1):146. doi: 10.1038/s41467-020-20161-9 33420015 PMC7794384

[34] Leontiou I, Davies T, Clark I, Aktar K, Suresh AP, Abad MA, et al. Bub1 kinase acts as a signalling hub for the entire Cryptococcus neoformans spindle assembly checkpoint pathway. bioRxiv. 2022:2022.09.21.508923.

[35] Aktar K, Davies T, Leontiou I, Clark I, Spanos C, Wallace E, et al. Conserved signalling functions for Mps1, Mad1 and Mad2 in the Cryptococcus neoformans spindle checkpoint. PLoS Genet. 2024 Jun;20(6):e1011302. doi: 10.1371/journal.pgen.1011302 38829899 PMC11175454

[36] Sionov E, Lee H, Chang YC, Kwon-Chung KJ. Cryptococcus neoformans overcomes stress of azole drugs by formation of disomy in specific multiple chromosomes. PLoS Pathog. 2010 Apr 1;6(4):e1000848. doi: 10.1371/journal.ppat.1000848 20368972 PMC2848560

[37] Xu Z, Cetin B, Anger M, Cho US, Helmhart W, Nasmyth K, et al. Structure and function of the PP2A-shugoshin interaction. Mol Cell. 2009 Aug 28;35(4):426–41. doi: 10.1016/j.molcel.2009.06.031 19716788 PMC2749713

[38] Liu H, Jia L, Yu H. Phospho-H2A and cohesin specify distinct tension-regulated Sgo1 pools at kinetochores and inner centromeres. Current biology: CB. 2013 Oct 7;23(19):1927–33. doi: 10.1016/j.cub.2013.07.078 24055156

[39] Gutierrez-Caballero C, Cebollero LR, Pendas AM. Shugoshins: from protectors of cohesion to versatile adaptors at the centromere. Trends Genet. 2012 Jul;28(7):351–60. doi: 10.1016/j.tig.2012.03.003 22542109

[40] Marston AL. Shugoshins: tension-sensitive pericentromeric adaptors safeguarding chromosome segregation. Mol Cell Biol. 2015 Feb;35(4):634–48. doi: 10.1128/MCB.01176-14 25452306 PMC4301718

[41] Kitajima TS, Kawashima SA, Watanabe Y. The conserved kinetochore protein shugoshin protects centromeric cohesion during meiosis. Nature. 2004 Feb 5;427(6974):510–7. doi: 10.1038/nature02312 14730319

[42] Ohkusu M, Raclavsky V, Takeo K. Induced synchrony in Cryptococcus neoformans after release from G2-arrest. Antonie Van Leeuwenhoek. 2004 Jan;85(1):37–44. doi: 10.1023/B:ANTO.0000020272.19569.15 15031662

[43] Hindriksen S, Lens SMA, Hadders MA. The Ins and Outs of Aurora B Inner Centromere Localization. Front Cell Dev Biol. 2017;5:112. doi: 10.3389/fcell.2017.00112 29312936 PMC5743930

[44] Varshney N, Som S, Chatterjee S, Sridhar S, Bhattacharyya D, Paul R, et al. Spatio-temporal regulation of nuclear division by Aurora B kinase Ipl1 in Cryptococcus neoformans. PLoS Genet. 2019 Feb;15(2):e1007959. doi: 10.1371/journal.pgen.1007959 30763303 PMC6392335

[45] Tatchell K, Makrantoni V, Stark MJ, Robinson LC. Temperature-sensitive ipl1-2/Aurora B mutation is suppressed by mutations in TOR complex 1 via the Glc7/PP1 phosphatase. Proc Natl Acad Sci U S A. 2011 Mar 8;108(10):3994–9. doi: 10.1073/pnas.1014406108 21368139 PMC3053998

[46] Lesage B, Qian J, Bollen M. Spindle checkpoint silencing: PP1 tips the balance. Current biology: CB. 2011 Nov 8;21(21):R898–903. doi: 10.1016/j.cub.2011.08.063 22075433

[47] Rosenberg JS, Cross FR, Funabiki H. KNL1/Spc105 recruits PP1 to silence the spindle assembly checkpoint. Current biology: CB. 2011 Jun 7;21(11):942–7. doi: 10.1016/j.cub.2011.04.011 21640906 PMC3109435

[48] Meadows JC, Shepperd LA, Vanoosthuyse V, Lancaster TC, Sochaj AM, Buttrick GJ, et al. Spindle checkpoint silencing requires association of PP1 to both Spc7 and kinesin-8 motors. Dev Cell. 2011 Jun 14;20(6):739–50. doi: 10.1016/j.devcel.2011.05.008 21664573 PMC3792844

[49] Espeut J, Cheerambathur DK, Krenning L, Oegema K, Desai A. Microtubule binding by KNL-1 contributes to spindle checkpoint silencing at the kinetochore. The Journal of cell biology. 2012 Feb 20;196(4):469–82. doi: 10.1083/jcb.201111107 22331849 PMC3284002

[50] Vanoosthuyse V, Hardwick KG. A novel protein phosphatase 1-dependent spindle checkpoint silencing mechanism. Current biology: CB. 2009 Jul 28;19(14):1176–81. doi: 10.1016/j.cub.2009.05.060 19592249 PMC2791888

[51] Nijenhuis W, Vallardi G, Teixeira A, Kops GJ, Saurin AT. Negative feedback at kinetochores underlies a responsive spindle checkpoint signal. Nat Cell Biol. 2014 Dec;16(12):1257–64. doi: 10.1038/ncb3065 25402682 PMC6485516

[52] Guacci V, Koshland D, Strunnikov A. A direct link between sister chromatid cohesion and chromosome condensation revealed through the analysis of MCD1 in S. cerevisiae. Cell. 1997 Oct 3;91(1):47–57. 9335334 10.1016/s0092-8674(01)80008-8 PMC2670185

[53] Michaelis C, Ciosk R, Nasmyth K. Cohesins: chromosomal proteins that prevent premature separation of sister chromatids. Cell. 1997 Oct 3;91(1):35–45. doi: 10.1016/s0092-8674(01)80007-6 9335333

[54] Hwang LH, Lau LF, Smith DL, Mistrot CA, Hardwick KG, Hwang ES, et al. Budding yeast Cdc20: a target of the spindle checkpoint. Science. 1998 Feb 13;279(5353):1041–4. doi: 10.1126/science.279.5353.1041 9461437

[55] Arras SD, Chitty JL, Blake KL, Schulz BL, Fraser JA. A genomic safe haven for mutant complementation in Cryptococcus neoformans. PLoS One. 2015;10(4):e0122916. doi: 10.1371/journal.pone.0122916 25856300 PMC4391909

[56] Roy B, Han SJY, Fontan AN, Jema S, Joglekar AP. Aurora B phosphorylates Bub1 to promote spindle assembly checkpoint signaling. Current biology: CB. 2022 Jan 10;32(1):237–47 e6. doi: 10.1016/j.cub.2021.10.049 34861183 PMC8752509

[57] Audett MR, Johnson EL, McGory JM, Barcelos DM, Szalai EO, Przewloka MR, et al. The microtubule- and PP1-binding activities of Drosophila melanogaster Spc105 control the kinetics of SAC satisfaction. Mol Biol Cell. 2022 Jan 1;33(1):ar1. doi: 10.1091/mbc.E21-06-0307-T 34705493 PMC8886820

[58] Verzijlbergen KF, Nerusheva OO, Kelly D, Kerr A, Clift D, de Lima Alves F, et al. Shugoshin biases chromosomes for biorientation through condensin recruitment to the pericentromere. Elife. 2014;3:e01374. doi: 10.7554/eLife.01374 24497542 PMC3910079

[59] Peplowska K, Wallek AU, Storchova Z. Sgo1 regulates both condensin and Ipl1/Aurora B to promote chromosome biorientation. PLoS Genet. 2014 Jun;10(6):e1004411. doi: 10.1371/journal.pgen.1004411 24945276 PMC4063673

[60] Boyarchuk Y, Salic A, Dasso M, Arnaoutov A. Bub1 is essential for assembly of the functional inner centromere. The Journal of cell biology. 2007 Mar 26;176(7):919–28. doi: 10.1083/jcb.200609044 17389228 PMC2064078

[61] Kiburz BM, Reynolds DB, Megee PC, Marston AL, Lee BH, Lee TI, et al. The core centromere and Sgo1 establish a 50-kb cohesin-protected domain around centromeres during meiosis I. Genes Dev. 2005 Dec 15;19(24):3017–30. doi: 10.1101/gad.1373005 16357219 PMC1315405

[62] Yamagishi Y, Sakuno T, Shimura M, Watanabe Y. Heterochromatin links to centromeric protection by recruiting shugoshin. Nature. 2008 Sep 11;455(7210):251–5. doi: 10.1038/nature07217 18716626

[63] Nogueira C, Kashevsky H, Pinto B, Clarke A, Orr-Weaver TL. Regulation of centromere localization of the Drosophila Shugoshin MEI-S332 and sister-chromatid cohesion in meiosis. G3 (Bethesda). 2014 Jul 31;4(10):1849–58. doi: 10.1534/g3.114.012823 25081981 PMC4199692

[64] Resnick TD, Satinover DL, MacIsaac F, Stukenberg PT, Earnshaw WC, Orr-Weaver TL, et al. INCENP and Aurora B promote meiotic sister chromatid cohesion through localization of the Shugoshin MEI-S332 in Drosophila. Dev Cell. 2006 Jul;11(1):57–68. doi: 10.1016/j.devcel.2006.04.021 16824953 PMC7115953

[65] Wu F, Akbar H, Wang C, Yuan X, Dou Z, Mullen M, et al. Sgo1 interacts with CENP-A to guide accurate chromosome segregation in mitosis. J Mol Cell Biol. 2024 Apr 4;15(10). doi: 10.1093/jmcb/mjad061 37777834 PMC11181942

[66] Mishra PK, Thapa KS, Chen P, Wang S, Hazbun TR, Basrai MA. Budding yeast CENP-A(Cse4) interacts with the N-terminus of Sgo1 and regulates its association with centromeric chromatin. Cell Cycle. 2018;17(1):11–23. doi: 10.1080/15384101.2017.1380129 28980861 PMC5815428

[67] Luo J, Deng X, Buehl C, Xu X, Kuo MH. Identification of Tension Sensing Motif of Histone H3 in Saccharomyces cerevisiae and Its Regulation by Histone Modifying Enzymes. Genetics. 2016 Nov;204(3):1029–43. doi: 10.1534/genetics.116.192443 27672091 PMC5105839

[68] Ng TM, Lenstra TL, Duggan N, Jiang S, Ceto S, Holstege FC, et al. Kinetochore function and chromosome segregation rely on critical residues in histones H3 and H4 in budding yeast. Genetics. 2013 Nov;195(3):795–807. doi: 10.1534/genetics.113.152082 24037263 PMC3813865

[69] Luo J, Xu X, Hall H, Hyland EM, Boeke JD, Hazbun T, et al. Histone h3 exerts a key function in mitotic checkpoint control. Mol Cell Biol. 2010 Jan;30(2):537–49. doi: 10.1128/MCB.00980-09 19917722 PMC2798460

[70] Wang X, Yang Y, Duan Q, Jiang N, Huang Y, Darzynkiewicz Z, et al. sSgo1, a major splice variant of Sgo1, functions in centriole cohesion where it is regulated by Plk1. Dev Cell. 2008 Mar;14(3):331–41. doi: 10.1016/j.devcel.2007.12.007 18331714 PMC2279080

[71] Davidson RC, Cruz MC, Sia RA, Allen B, Alspaugh JA, Heitman J. Gene disruption by biolistic transformation in serotype D strains of Cryptococcus neoformans. Fungal Genet Biol. 2000 Feb;29(1):38–48. doi: 10.1006/fgbi.1999.1180 10779398

[72] Schindelin J, Arganda-Carreras I, Frise E, Kaynig V, Longair M, Pietzsch T, et al. Fiji: an open-source platform for biological-image analysis. Nat Methods. 2012 Jun 28;9(7):676–82. doi: 10.1038/nmeth.2019 22743772 PMC3855844

[73] Orner EP, Zhang P, Jo MC, Bhattacharya S, Qin L, Fries BC. High-Throughput Yeast Aging Analysis for Cryptococcus (HYAAC) microfluidic device streamlines aging studies in Cryptococcus neoformans. Commun Biol. 2019;2:256. doi: 10.1038/s42003-019-0504-5 31312725 PMC6620289

[74] Crane MM, Clark IB, Bakker E, Smith S, Swain PS. A microfluidic system for studying ageing and dynamic single-cell responses in budding yeast. PLoS One. 2014;9(6):e100042. doi: 10.1371/journal.pone.0100042 24950344 PMC4065030

[75] Granados AA, Pietsch JMJ, Cepeda-Humerez SA, Farquhar IL, Tkacik G, Swain PS. Distributed and dynamic intracellular organization of extracellular information. Proc Natl Acad Sci U S A. 2018 Jun 5;115(23):6088–93. doi: 10.1073/pnas.1716659115 29784812 PMC6003323

[76] Granados AA, Crane MM, Montano-Gutierrez LF, Tanaka RJ, Voliotis M, Swain PS. Distributing tasks via multiple input pathways increases cellular survival in stress. Elife. 2017 May 17;6. 10.7554/eLife.21415 PMC5464774 28513433

[77] Pietsch JMJ, Munoz AF, Adjavon DA, Farquhar I, Clark IBN, Swain PS. Determining growth rates from bright-field images of budding cells through identifying overlaps. Elife. 2023 Jul 7;12. doi: 10.7554/eLife.79812 37417869 PMC10371227

[78] Cai L, Dalal CK, Elowitz MB. Frequency-modulated nuclear localization bursts coordinate gene regulation. Nature. 2008 Sep 25;455(7212):485–90. doi: 10.1038/nature07292 18818649 PMC2695983

[79] Hao N O’Shea EK Signal-dependent dynamics of transcription factor translocation controls gene expression. Nat Struct Mol Biol. 2011 Dec 18;19(1):31–9. doi: 10.1038/nsmb.2192 22179789 PMC3936303

[80] Lin Y, Sohn CH, Dalal CK, Cai L, Elowitz MB. Combinatorial gene regulation by modulation of relative pulse timing. Nature. 2015 Nov 5;527(7576):54–8. doi: 10.1038/nature15710 26466562 PMC4870307

[81] de Jonge WJ, Brok M, Kemmeren P, Holstege FCP. An Optimized Chromatin Immunoprecipitation Protocol for Quantification of Protein-DNA Interactions. STAR Protoc. 2020 Jun 19;1(1):100020. doi: 10.1016/j.xpro.2020.100020 32685929 PMC7357673

[82] Mitra S, Gomez-Raja J, Larriba G, Dubey DD, Sanyal K. Rad51-Rad52 mediated maintenance of centromeric chromatin in Candida albicans. PLoS Genet. 2014 Apr;10(4):e1004344. doi: 10.1371/journal.pgen.1004344 24762765 PMC3998917

[83] Sievers F, Wilm A, Dineen D, Gibson TJ, Karplus K, Li W, et al. Fast, scalable generation of high-quality protein multiple sequence alignments using Clustal Omega. Mol Syst Biol. 2011 Oct 11;7:539. doi: 10.1038/msb.2011.75 21988835 PMC3261699

[84] Waterhouse AM, Procter JB, Martin DM, Clamp M, Barton GJ. Jalview Version 2—a multiple sequence alignment editor and analysis workbench. Bioinformatics. 2009 May 1;25(9):1189–91. doi: 10.1093/bioinformatics/btp033 19151095 PMC2672624

[85] Mohr L, Buheitel J, Schockel L, Karalus D, Mayer B, Stemmann O. An Alternatively Spliced Bifunctional Localization Signal Reprograms Human Shugoshin 1 to Protect Centrosomal Instead of Centromeric Cohesin. Cell Rep. 2015 Sep 29;12(12):2156–68. doi: 10.1016/j.celrep.2015.08.045 26365192

[86] Salic A, Waters JC, Mitchison TJ. Vertebrate shugoshin links sister centromere cohesion and kinetochore microtubule stability in mitosis. Cell. 2004 Sep 3;118(5):567–78. doi: 10.1016/j.cell.2004.08.016 15339662

[87] Tashiro S, Handa T, Matsuda A, Ban T, Takigawa T, Miyasato K, et al. Shugoshin forms a specialized chromatin domain at subtelomeres that regulates transcription and replication timing. Nat Commun. 2016 Jan 25;7:10393. doi: 10.1038/ncomms10393 26804021 PMC4737732

