# Supplementary figures and images for: The dependence of shugoshin on Bub1-kinase activity is dispensable for the maintenance of spindle assembly checkpoint response in Cryptococcus neoformans
Source: PLoS Genet. 2025 Jan 13;21(1):e1011552. doi: 10.1371/journal.pgen.1011552 (PMC11774493; doi:10.1371/journal.pgen.1011552)

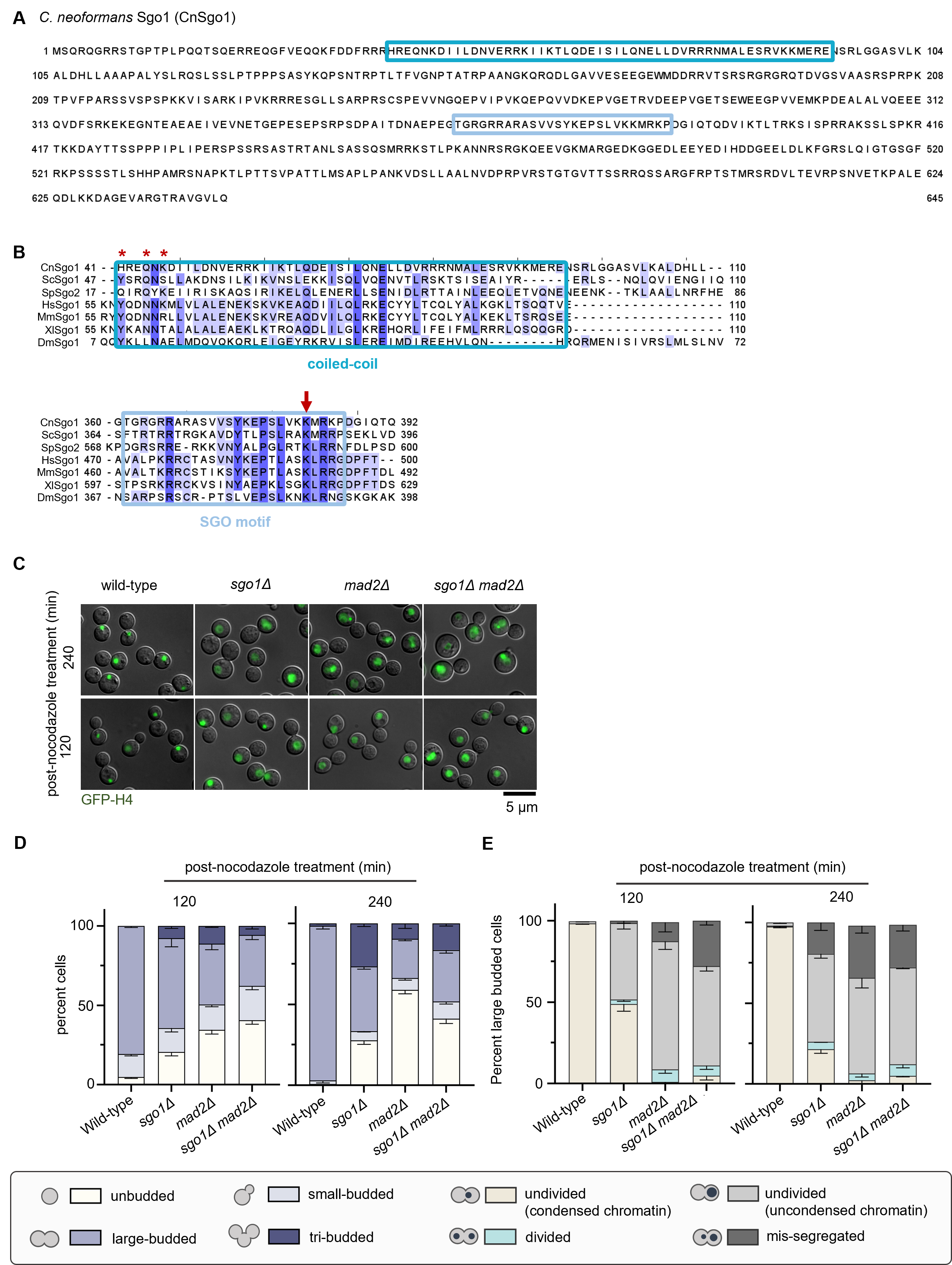

Supplement: S1 Fig — (A) The protein sequence of C. neoformans Sgo1. (B) Multiple sequence alignment (MSA) of the CnSgo1 N-terminal coiled-coil and the C-terminal SGO motif with vertebrate and invertebrate species (Cn- C. neoformans; Sc- Saccharomyces cerevisiae; Sp- Schizosaccharomyces pombe; Hs- Homo sapiens; Mm- Mus musculus; Xl- Xenopus laevis; Dm- Drosophila melanogaster). Multiple sequence alignment of Sgo1 homologs was performed using Clustal Omega [83], and the alignment was formatted using Jalview 2 [84]. *Represent amino acid residues involved in PP2A binding. The dark blue circle represents the key residue (K382 in C. neoformans) required for Bub1-mediated kinetochore proximal centromere localization of shugoshin. The teal blue colored box represents the coiled-coil domain. The light blue colored box represents the conserved basic SGO motif. (C) Microscopic images of GFP-H4 in CNVY108 (SGO1 MAD2), CNSD117 (sgo1Δ), SHR866 (mad2Δ), CNSD148 (sgo1Δ mad2Δ) treated with nocodazole (1 μg/mL) for 120- and 240-min. Scale bar, 5 μm. (D) Bar graphs representing the proportion of unbudded, small-budded, large-budded, and tri-budded cells. N = 3, n>100 cells counted in each experiment. (E) Bar graphs representing the proportion of large-budded cells of indicated phenotypes, N = 3, n>100 cells counted in each experiment. (TIF) [file pgen.1011552.s001.tif]

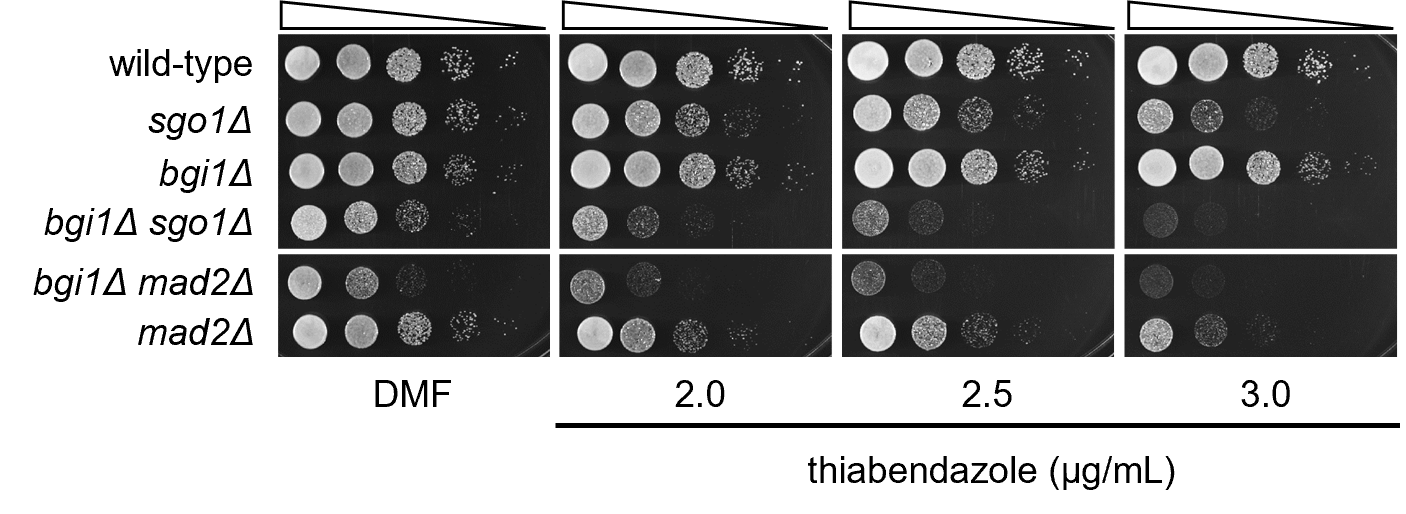

Supplement: S2 Fig — A ten-fold serial dilution spotting assay to score for the sensitivity of the wild-type CNVY108 (SGO1 BGI1 GFP-H4), CNSD117 (sgo1Δ GFP-H4), SHR741 (mad2Δ GFP-H4), SHR830 (bgi1Δ GFP-H4), CNSD148 (sgo1Δ mad2Δ GFP-H4) and CNSD163 (sgo1Δ bgi1Δ GFP-H4) to thiabendazole. No drug represents DMF (Dimethyl formamide) only. The plates were incubated at 30°C for 24 h. (TIF) [file pgen.1011552.s002.tif]

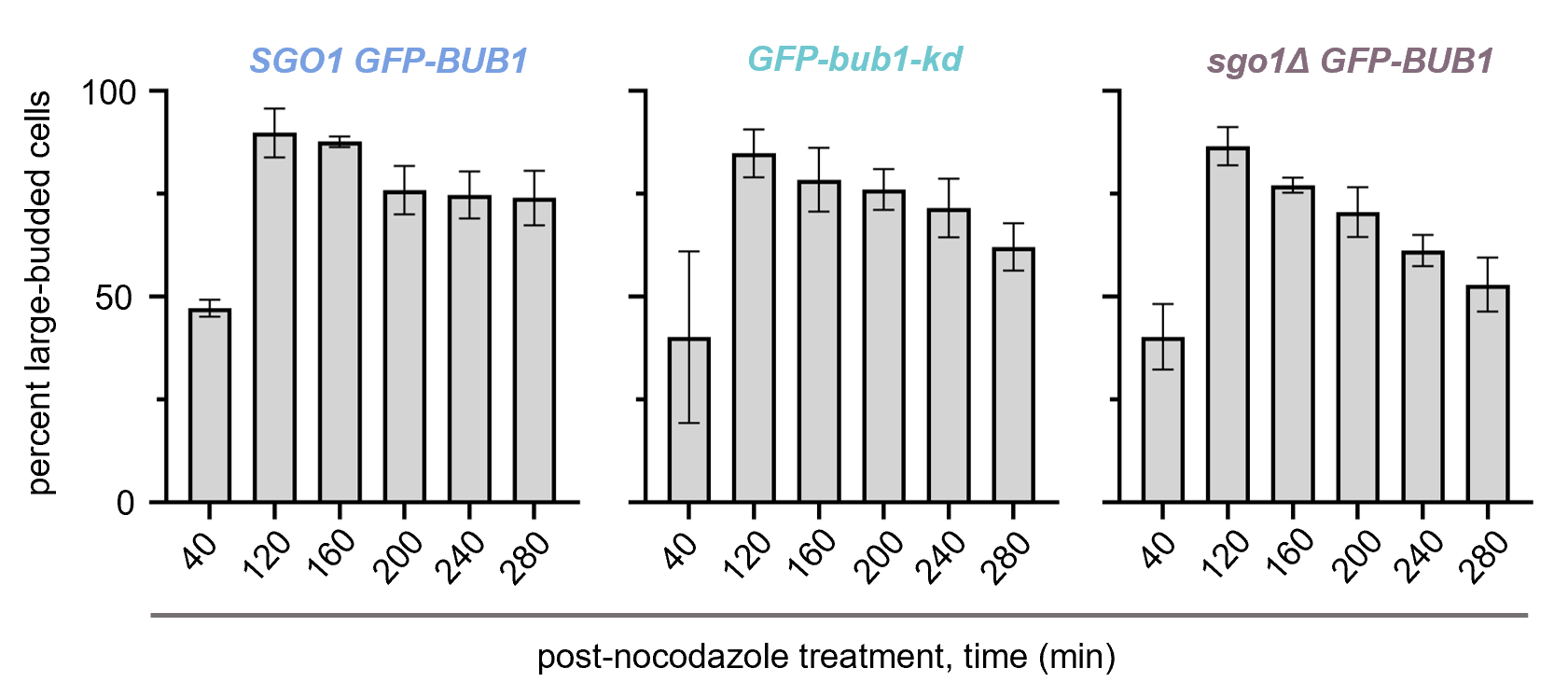

Supplement: S3 Fig — Bar graphs representing the proportion of large-budded cells obtained post nocodazole treatment in the indicated strains, N = 3, n>100, cells counted in each experiment. Errors bars represent SD. (TIF) [file pgen.1011552.s003.tif]

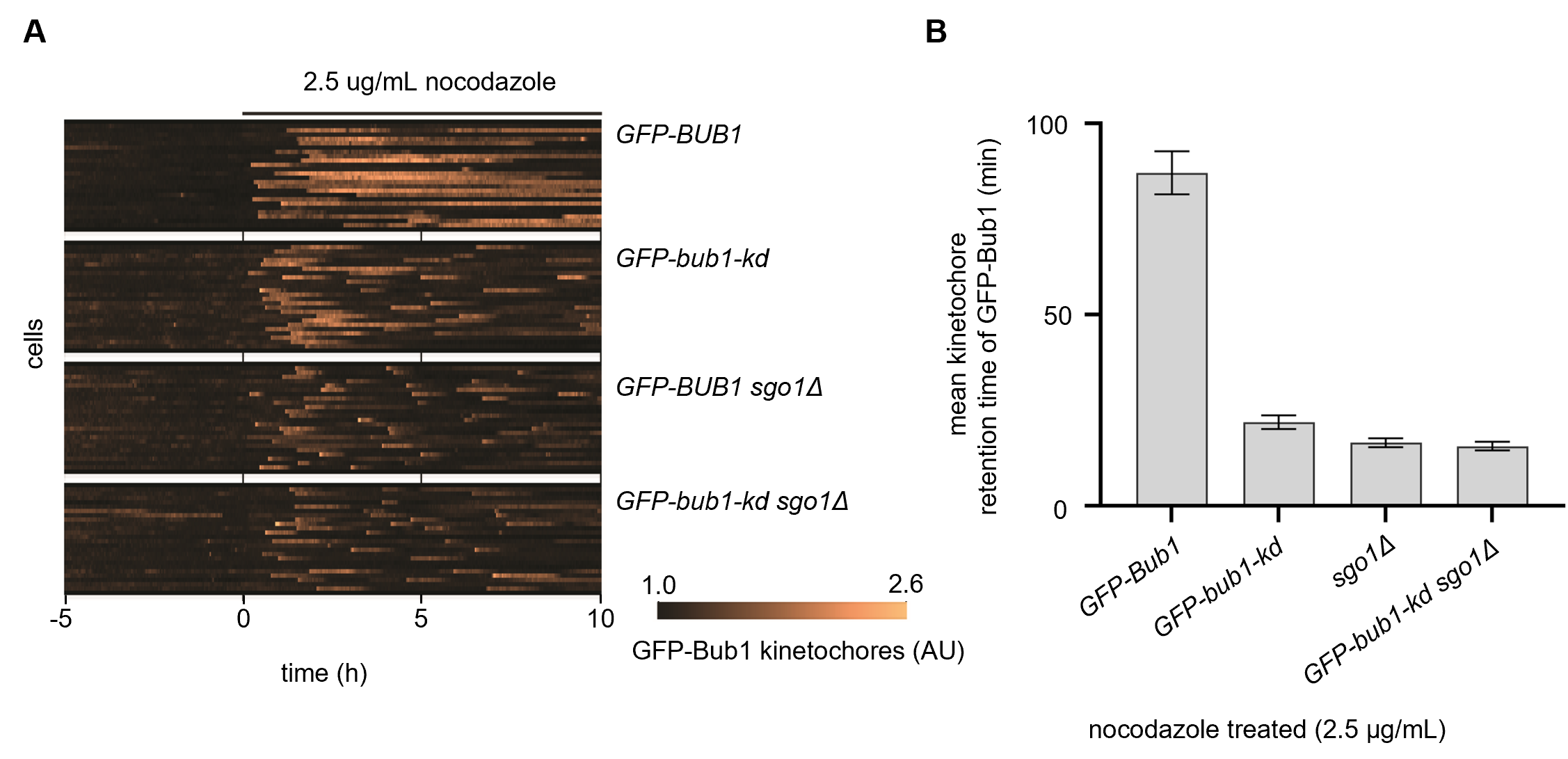

Supplement: S4 Fig — (A) Microfluidics assay to determine the retention time of GFP-Bub1 at kinetochores in response to nocodazole treatment. Temporal heat maps of 30 randomly selected cells are shown. The heat map represents the changes in the kinetochore localization signal of GFP-Bub1 over time. Each bright track on the y-axis of the heat map represents GFP-Bub1 signals from an individual cell (median of the brightest 5 pixels in each cell divided by the overall cell median brightness). The length of each bright track along the x-axis represents the time (min). The time of addition of nocodazole (2.5 μg/mL) is considered as 0 hr. Images are taken every 2 min for 10 h. Assay was performed using IL102 (GFP-BUB1), IL143 (GFP-bub1-kd), CNSD176 (GFP-BUB1 sgo1Δ), and CNSD173 (GFP-bub1-kd sgo1Δ) strains. (B) Bar graphs representing the quantitative analysis of GFP-Bub1 retention time at kinetochores obtained from microfluidics assays in the above-indicated strains. n = 24, error bars represent SEM. (TIF) [file pgen.1011552.s004.tif]

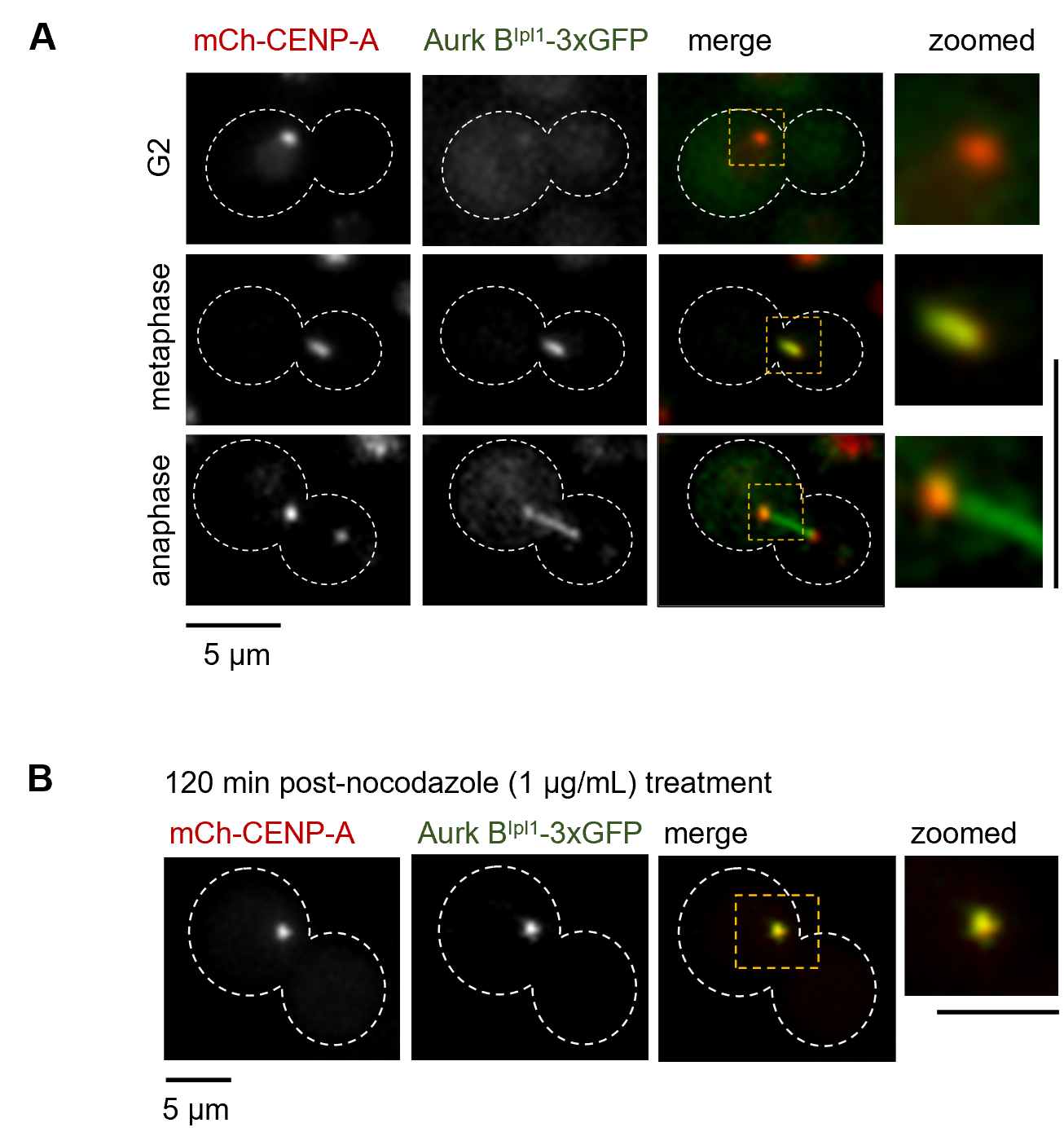

Supplement: S5 Fig — (A) Microscopic images of Aurora BIpl1-3xGFP localized with a kinetochore protein mCherry-CENP-A at G2, metaphase, and anaphase stages of the cell cycle. (B) Colocalization of Aurora BIpl1-3xGFP with mCherry-CENP-A when treated with nocodazole (1 μg/mL) for 120 min. Scale bar, 5 μm. (TIF) [file pgen.1011552.s005.tif]

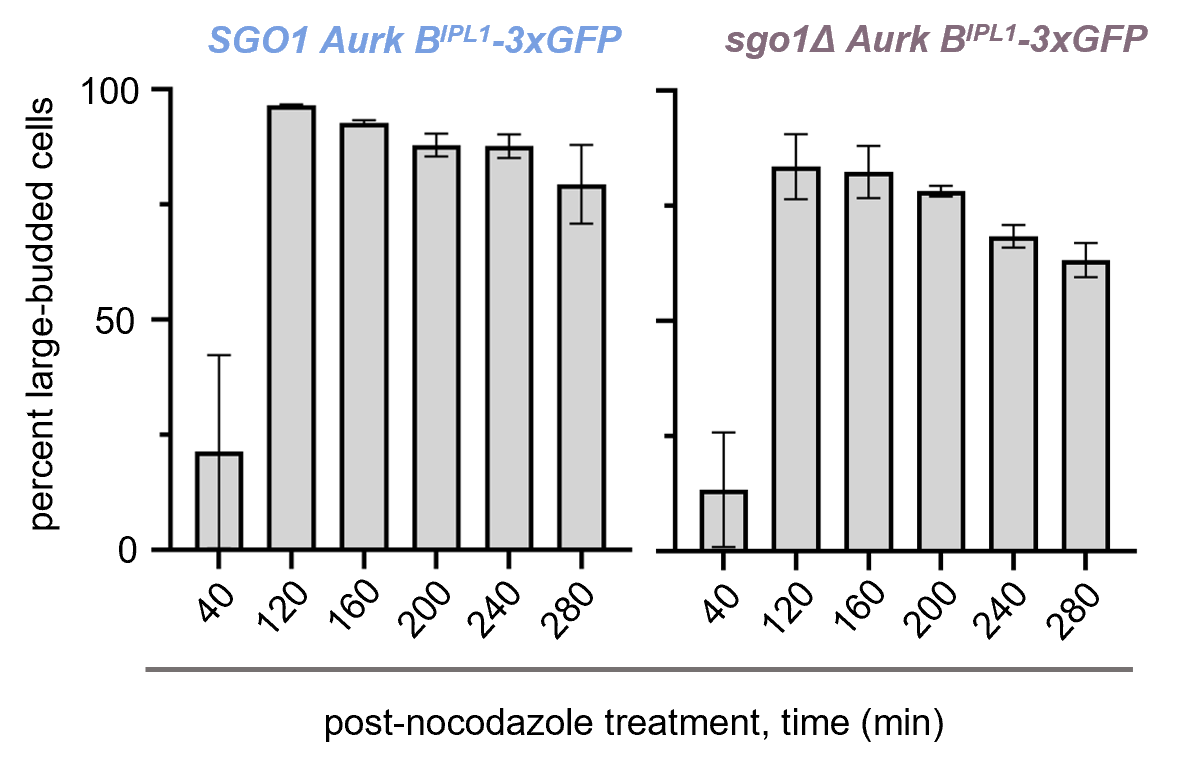

Supplement: S6 Fig — Bar graphs representing the proportion of large-budded cells obtained post nocodazole treatment in the indicated strains, N = 3, n>100, cells counted in each experiment. Errors bars represent SD. (TIF) [file pgen.1011552.s006.tif]

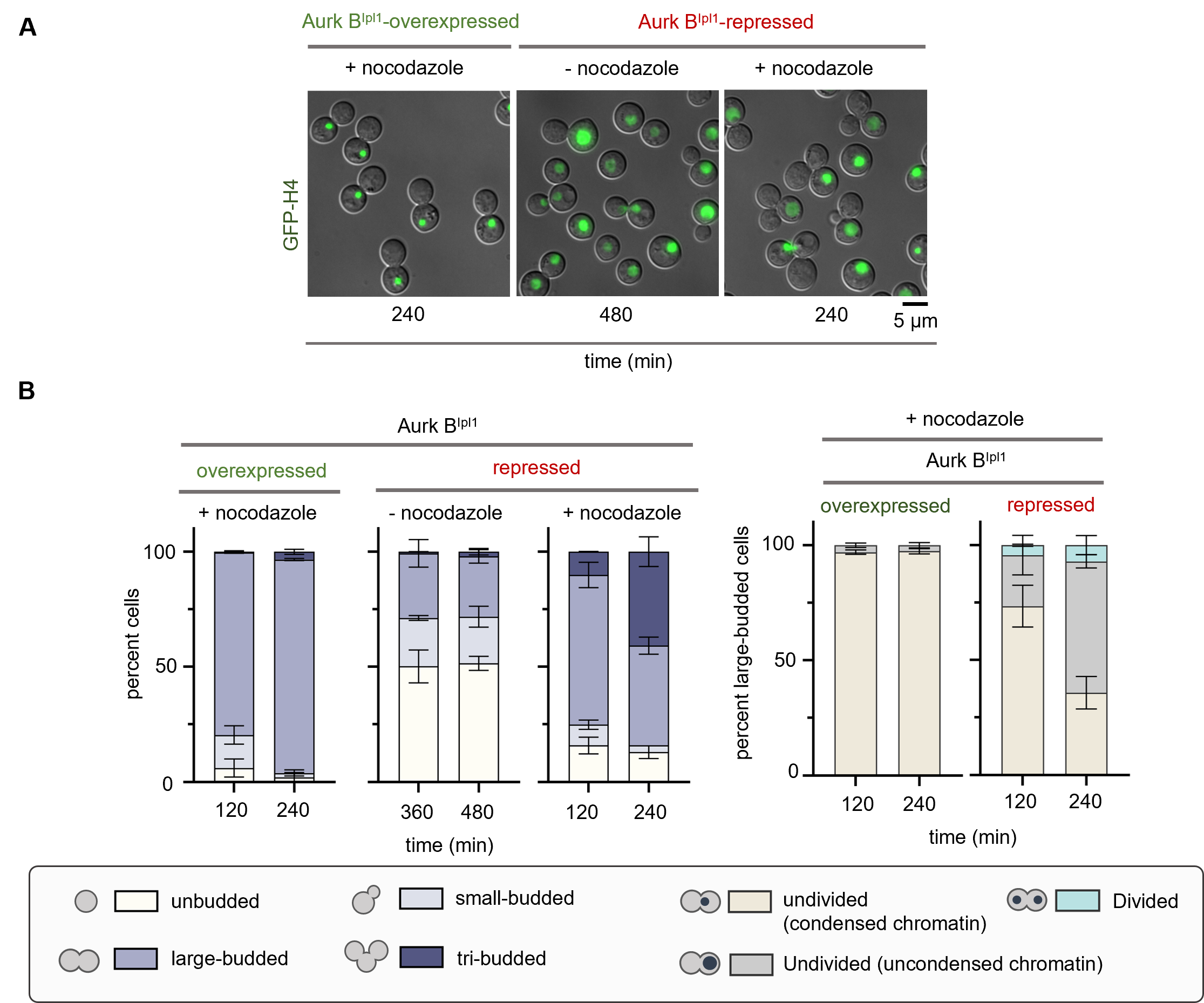

Supplement: S7 Fig — (A) Microscopic images of CNNV104 (GFP-H4 GAL7-AURORA BIPL1) grown in permissive and non-permissive media in the presence and absence of nocodazole. Scale bar, 5 μm. Representative images of cells treated with nocodazole and depleted of Aurora BIpl1 for 240 and 480 min were shown. (B) Left, bar graphs representing the proportion of unbudded, small-budded, large-budded, and tri-budded cells. Right, bar graphs representing the proportion of large-budded cells of indicated phenotype, N = 2, n>100 cells counted for each experiment. (TIF) [file pgen.1011552.s007.tif]

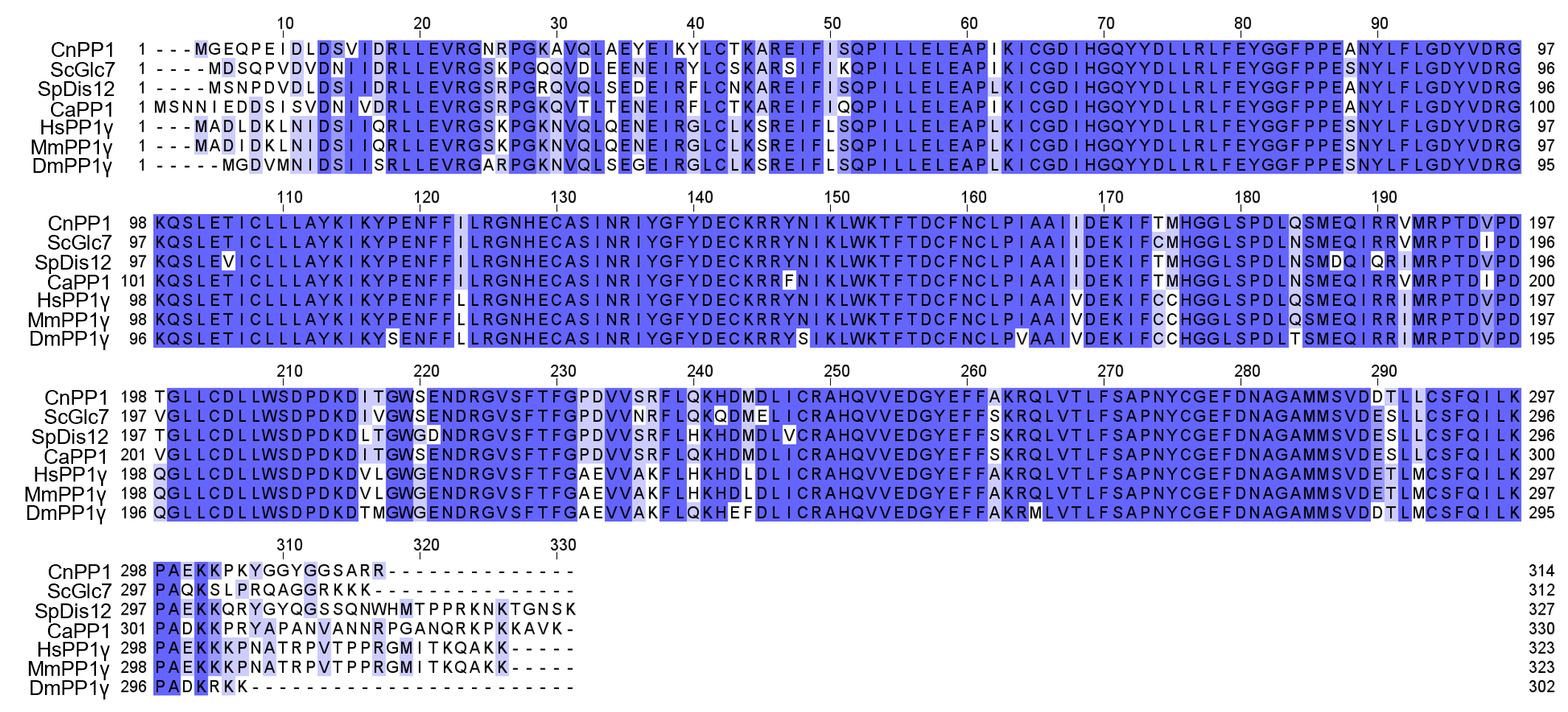

Supplement: S8 Fig — Multiple sequence alignment of PP1 homolog of C. neoformans (CNAG_03706) with vertebrate and invertebrate species (Cn- C. neoformans; Sc- S. cerevisiae; Sp- S. pombe; Ca- Candida albicans; Hs- H. sapiens; Mm- M. musculus; Dm- D. melanogaster) was performed using Clustal Omega [83] and the alignment was formatted using Jalview 2 [84]. Highly conserved regions are shaded in dark blue. (TIF) [file pgen.1011552.s008.tif]

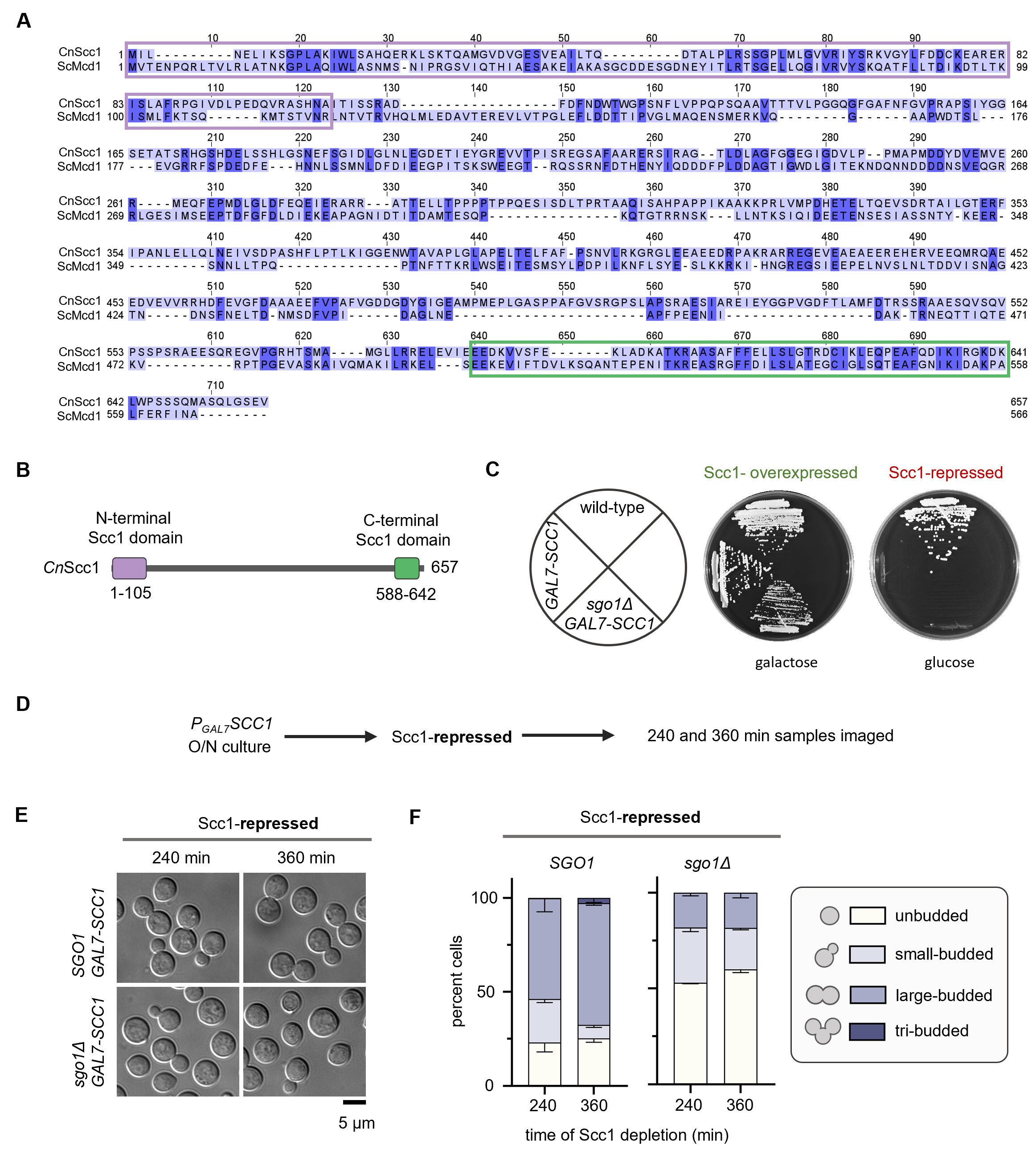

Supplement: S9 Fig — (A) Pairwise sequence alignment of Scc1 homologs of C. neoformans (CNAG_01023) and S. cerevisiae by Clustal Omega [83], and formatted using Jalview 2 [84]. Highly conserved regions are shaded in dark blue. The two domains corresponding to N- and C-terminal Scc1 domains (E-values of 2.0e-31 and 2.2e-15, respectively) of CnScc1 are highlighted in violet and green boxes. (B) Schematic of the domains present in C. neoformans Scc1 protein. (C) Plate photographs of strains expressing Scc1 under the regulatable GAL7 promoter grown in media containing glucose (non-permissive) and galactose (permissive). (D) Schematic to check the response of Scc1 repression in SGO1 and sgo1Δ backgrounds. (E) Microscopic images of GFP-H4 tagged CNSD181 (GAL7-3xFLAG-SCC1) and CNSD182 (sgo1Δ GAL7-3xFLAG-SCC1) strains depleted of Scc1. Representative images of cells depleted of Scc1 for 4 and 6 h were shown. Scale bar, 5 μm. (F) Bar graphs representing the percentage of unbudded, small-budded, large-budded, and tri-budded cells scored. N = 2, n>100 cells for each experiment, error bars represent SD. (TIF) [file pgen.1011552.s009.tif]

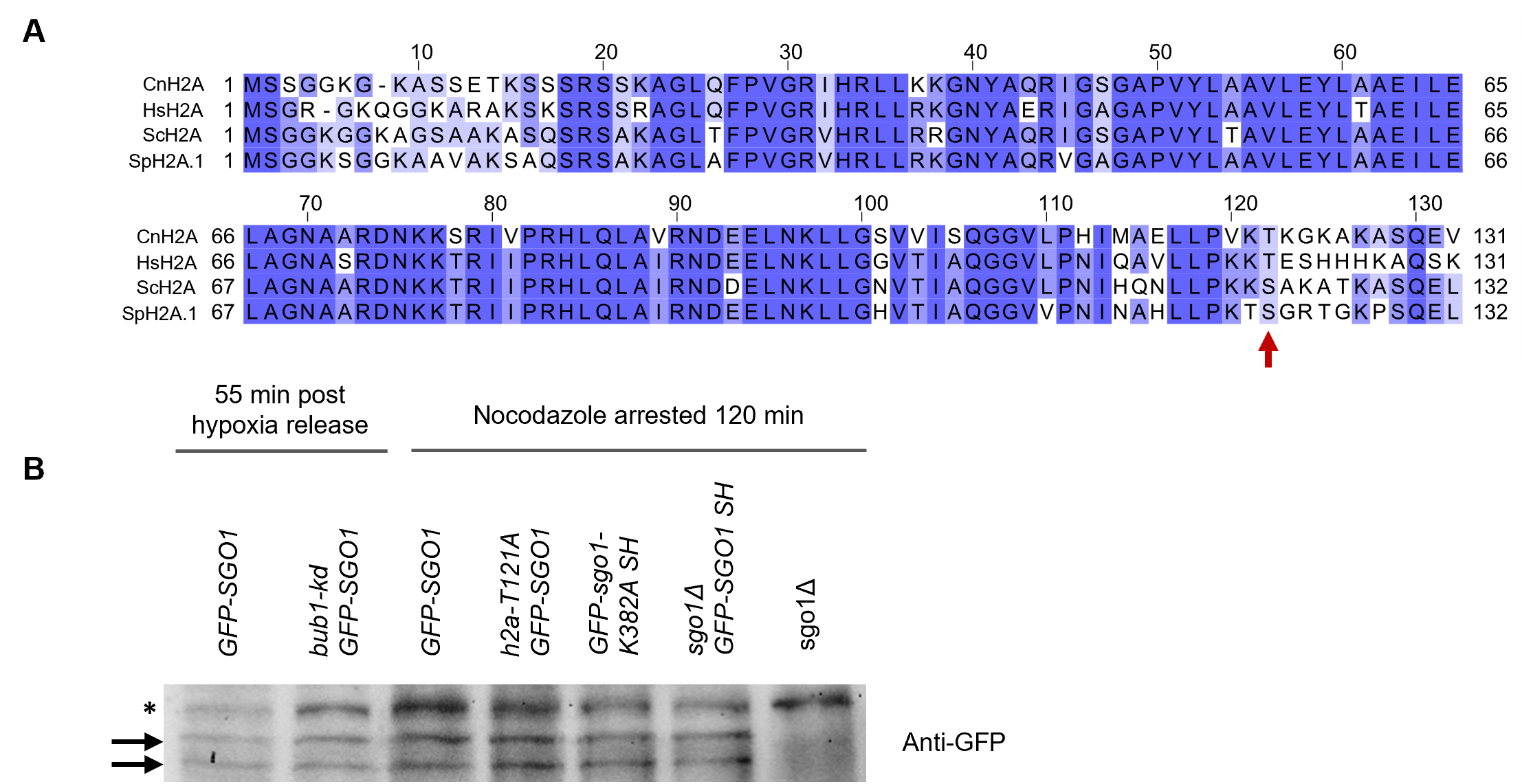

Supplement: S10 Fig — (A) Multiple sequence alignment of H2A homologs obtained from C. neoformans (Cn), S. cerevisiae (Sc), S. pombe (Sp) and H. sapiens (Hs). The alignment was performed using Clustal Omega [83] and formatted using Jalview 2 [84]. The dark blue shaded regions represent highly conserved amino acid residues. Arrow indicates the conserved T121/S120 residue phosphorylated by Bub1. (B) Western blot showing the expression levels of GFP-Sgo1 at metaphase in the indicated strains. * Indicates non-specific band used as a loading control. Arrows indicate two bands of GFP-Sgo1. sgo1Δ lane represents no tag control. (TIF) [file pgen.1011552.s010.tif]

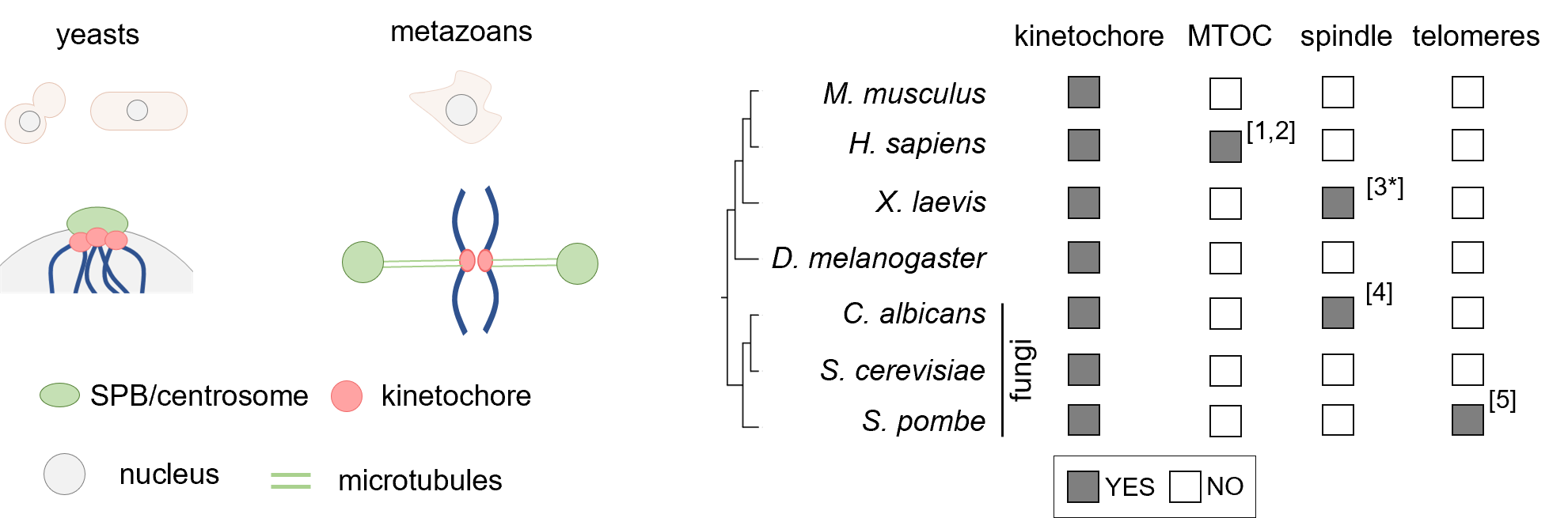

Supplement: S11 Fig — Left, schematic highlighting the spatial positioning of SPB/centrosome and centromeres in yeasts and metazoans. Right, localization of shugoshin with respect to the kinetochore, MTOCs (centrosomes or SPBs), spindle MTs, and telomeres. Filled and open squares represent the presence or absence of shugoshin at the indicated sub-cellular locations. The data was compiled from reviews [39, 40]. [1,2] [70, 85], [3*] [86], this study has shown that the purified N-terminal region of shugoshin is capable of binding to MTs in vitro. [4] [25] and [5] [87]. (TIF) [file pgen.1011552.s011.tif]
