# Supplementary material for: The dependence of shugoshin on Bub1-kinase activity is dispensable for the maintenance of spindle assembly checkpoint response in Cryptococcus neoformans
Source: PLoS Genet. 2025 Jan 13;21(1):e1011552. doi: 10.1371/journal.pgen.1011552 (PMC11774493; doi:10.1371/journal.pgen.1011552)
Supplement: S1 Table — (DOCX) [file pgen.1011552.s012.docx]

**Table 1: List of strains**

| **Strain** | **Genotype** | **Source** |
| --- | --- | --- |
| H99 | *MATα* (wild-type) | [88] |
| CNVY101 | *MATa::mCherry-CENP-A-NEO (pLKB74)* | [30] |
| CNVY108 | *MATα::GFP-H4-NAT (pVY3)* | [30] |
| SHR741 | *MATα::GFP-H4-NAT (pVY3) MAD2::mad2-NEO* | [33] |
| SHR830 | *MATα::GFP-H4-NAT (pVY3) BGI1::bgi1Δ-HygB* | [33] |
| IL143 | *MATα::BUB1::bub1Δ-NAT HISp-GFP-bub1-kd-HYGB (chromosome 3, safe haven p37)* | [34] |
| IL102 | *MATα::BUB1::bub1Δ-NAT HISp-GFP-bub1-HygB (chromosome 3, safe haven p37)* | [34] |
| IL089 | *MATα::MAD1::mad1Δ HISp:GFP-Bub1: HygB (chromosome 3, safe haven p37)* | [34] |
| CNNV113 | *MATα:: AURK B^IPL1^:: AURK B^IPL1^p- AURK B^IPL1^-3xGFP-NEO* | [44] |
| CNNV104 | *MATα:GFP-H4-NAT (pVY3),*  *AURK B^IPL1^::GAL7p- AURK B^IPL1^- HygB* | [44] |
| CNSD110 | *MATα::SGO1::sgo1Δ-NEO* | This Study |
| CNSD117 | *MATα::GFP-H4-NAT (pVY3) SGO1::sgo1Δ-NEO* | This Study |
| CNSD125 | *MATa::mCherry-CENP-A-NEO (pLKB74) SGO1::SGO1pr-GFP-SGO1-NAT (pSD10)* | This study |
| CNSD127 | *MATα::SGO1::SGO1p-GFP-SGO1-NAT (pSD10)* | This study |
| CNSD130 | *MATα::SGO1::SGO1p-GFP-SGO1-NAT (pSD10) TUB4::TUB4-mCherry-NEO* | This study |
| CNSD148 | *MATα::GFP-H4-NAT (pVY3) MAD2::mad2-NEO SGO1::sgo1Δ-NEO* | This study |
| CNSD163 | *MATα::GFP-H4-NAT (pVY3) BGI1::bgi1Δ- HygB SGO1::sgo1Δ-NEO* | This study |
| CNSD167 | *MATa::mCherry-CENP-A-NEO (pLKB74) SGO1::SGO1pr-GFP-SGO1-NAT (pSD10) CDC20p::GAL7p-3xFLAG-CDC20-HYGB* | This study |
| CNSD173 | *MATα::BUB1::bub1Δ-NAT HISp-GFP-bub1-kd- HygB (chromosome 3, safe haven p37) SGO1::sgo1Δ-NEO* | This study |
| CNSD176 | *MATα::BUB1::bub1Δ-NAT HISp-GFP-bub1- HygB (chromosome 3, safe haven p37) SGO1::sgo1Δ-NEO* | This study |
| CNSD180 | *MATa::mcherry-CENP-A-NEO PP1:: PP1p-GFP-PP1-NAT* (pKB004) *SGO1::sgo1Δ-HYGB* | This study |
| CNSD181 | *MATα::GFP-H4-NAT (pVY3) SCC1p::GAL7p-3xFLAG-SCC1- HygB* | This study |
| CNSD182 | *MATα::GFP-H4-NAT (pVY3) SCC1p::GAL7p-3xFLAG-SCC1- HygB SGO1::sgo1Δ-NEO* | This study |
| CNSD183 | *MATa::mCherry-CENP-A-NEO (pLKB74) SGO1::SGO1pr-GFP-SGO1-NAT (pSD10)*  *SCC1p::GAL7p-3xFLAG-SCC1- HygB* | This study |
| CNSD190 | *MATα::AURK B^IPL1^:: AURK B^IPL1^p- AURK B^IPL1^-3xGFP-NEO mCherry-CENP-A- HygB (pLKB71)* | This study |
| CNSD196 | *MATα:: AURK B^IPL1^:: AURK B^IPL1^p- AURK B^IPL1^-3xGFP-NEO mCherry-CENP-A-HygB (pLKB71) SGO1::sgo1Δ-NAT* | This study |
| CNSD205 | *MATα::BUB1::bub1Δ-NAT HISp-GFP-bub1- HygB (chromosome 3, safe haven p37) AURK B^IPL1^::GAL7-3xFLAG-AUR B^IPL1^-NEO* | This study |
| CNSD207 | *MATα::SGO1::sgo1Δ-NEO SGO1p-GFP-SGO1- HygB (pSD34) (SAFE HAVEN1, chrormosome1)* | This study |
| CNSD209 | *MATα::SGO1::sgo1Δ-NEO SGO1p-GFP-sgo1-K382A- HygB (pSD35) (SAFE HAVEN1, chrormosome1)* | This study |
| CNSD215 | *MATa::mCherry-CENP-A-NEO (pLKB74) SGO1::SGO1pr-GFP-SGO1-NAT (pSD10)*  *Bub1-kd-3xFLAG-HygB* (*pSD39*) | This study |
| CNSD218 | *MATα::SGO1::sgo1Δ-NEO SGO1p-GFP-sgo1-K382A- HygB (pSD35) (SAFE HAVEN1, chrormosome1)* | This study |
| CNSD219 | *MATα::SGO1::sgo1Δ-NEO SGO1p-GFP-sgo1-K382A- HygB (pSD35) (SAFE HAVEN1, chrormosome1) mCherry-CENP-A-NAT* (random integration) (*pSD41*) | This study |
| CNKB003 | *MATa::mcherry-CENP-A-NEO PP1:: PP1p-GFP-PP1-NAT* (pKB004) | This study |
| CNKB023 | *MATa::mcherry-CENP-A-NEO PP1:: PP1p-GFP-PP1-NAT* (pKB004) *AURK B^IPL1^::GAL7-3XFLAG-AURK B^IPL1^-NEO* | This study |
