# Supplementary material for: The dependence of shugoshin on Bub1-kinase activity is dispensable for the maintenance of spindle assembly checkpoint response in Cryptococcus neoformans
Source: PLoS Genet. 2025 Jan 13;21(1):e1011552. doi: 10.1371/journal.pgen.1011552 (PMC11774493; doi:10.1371/journal.pgen.1011552)
Supplement: S2 Table — (DOCX) [file pgen.1011552.s013.docx]

**Table 2: List of plasmids**

| **Plasmid** | **Description** | **Source** |
| --- | --- | --- |
| pVY7 | *H3p-GFP-NAT* from pCN19 cloned into pBSII using SacI-ApaI | [89] |
| pSH7G | *HygB-GAL7-GFP* pBlueScriptII KS(-) | [33] |
| pLKB71 | pXLI + *mCherry-CENP-A-HygB* | From Joseph Heitman, Duke University, US |
| pSD10 | *H3p* of pVY7 replaced with *SGO1p* (SacI/NcoI) + *SGO1* homology region (BamHI) | This study |
| pSD29 | *GAL7p of p*SH7G replaced with *SGO1p* (SpeI/BamHI)+*SGO1* 3’UTR (NheI/ApaI)+*SAFE HAVEN1* (ApaI) | This study |
| pSD34 | *SGO1* allele (HpaI/NheI) cloned into pSD29 | This study |
| pSD35 | *sgo1-K382A* allele (HpaI/NheI) cloned into pSD29 | This study |
| pSD39 | *GAL7-GFP of pSH7G replaced with bub1-kd* homology region harbouring *K1011R* and *D1149N* (SpeI/KpnI) | This study |
| pSD41 | pXLI + *mCherry-CENP-A-NAT* | This study |
| pKB004 | *H3p* of pVY7 replaced with *PP1p* (SacI/NcoI) +*PP1* homology region (SpeI) | This study |
