# Supplementary material for: The dependence of shugoshin on Bub1-kinase activity is dispensable for the maintenance of spindle assembly checkpoint response in Cryptococcus neoformans
Source: PLoS Genet. 2025 Jan 13;21(1):e1011552. doi: 10.1371/journal.pgen.1011552 (PMC11774493; doi:10.1371/journal.pgen.1011552)
Supplement: S3 Table — (DOCX) [file pgen.1011552.s014.docx]

**Table 3: List of primers**

| **Primer number** | **Sequence 5'-3'** | **Purpose** |
| --- | --- | --- |
| SDP1 | CCGATGAGCGTACACCACTTAC | *SGO1* deletion overlap primers |
| SDP2 | CCAGCTCACATCCTCGCAGCTGCTACGAGATTGAGACGAATG |  |
| SDP3 | CATTCGTCTCAATCTCGTAGCAGCTGCGAGGATGTGAGCTGG |  |
| SDP4 | CTCCCAACTTCCCCGCTTTTGGTTTATCTGTATTAACACGGAAGAG |  |
| SDP5 | CTCTTCCGTGTTAATACAGATAAACCAAAAGCGGGGAAGTTGGGAG |  |
| SDP6 | ATCAGAACGCACGTCGCAC |  |
| SDP7 | CAGGCAGAGGATTGGGAAGTC |  |
| SDP8 | CCAGTTCCTCAAAGAGCTTGAC | *SGO1* deletion confirmation primer |
| SDP20 | ACTGAGCTCACTATCTGCAAAATCGACTTG | *SGO1pr-GFP-SGO1* N-terminal tagging |
| SDP21 | CATGCCATGGCCTGTATTTACGCCTGGAG |  |
| SDP22 | CGCGGATCCATGTCCCAGAGGCAAGGCAG |  |
| SDP23 | CGCGGATCCGTTTCATCTCGACCACAGGTC |  |
| SDP24 | GGCACAAGCTGGAGTACAAC | Universal forward primer for tagging confirmation |
| SDP25 | CTTCAGGTTCAGCATTGTCTG |  |
| SDP27 | ACTGGGCCCACACGATGTTTTCCATCAG | *SAFE HAVEN1* cloning |
| SDP28 | ACTGGGCCCGGTCTCACAATCATCAACATC |  |
| SDP54 | AACAGTGATGGCAGCGTC | *TUB4-mCherry* C-terminal tagging overlap |
| SDP55 | CGCATCCAACGTCATCCAAG |  |
| VYP360 | CTTGTGAACAATGCTGATAGTGTGG |  |
| VYP361 | CTCGCCCTTGCTCACCATCTCCCCCCCAAAATCGATATAATCC |  |
| VYP362 | GGATTATATCGATTTTGGGGGGGAGATGGTGAGCAAGGGCGAG |  |
| VYP363 | CGAAGTGCGTACTTGAAAGAAGTCCAAGCTTGGTACCGAGCTC |  |
| VYP364 | GAGCTCGGTACCAAGCTTGGACTTCTTTCAAGTACGCACTTCG |  |
| VYP365 | TGATGATCTCCAAGCTCTTCAC |  |
| SDP108 | TTGTTGTTACCATCATCCTCTC | Universal confirmation Reverse primer for deletion cassettes |
| SDP110 | AGATCGACAACATGCGACC | *GAL7-3xFLAG-Cdc20* tagging overlap |
| SDP111 | CTGCAGATATCCATCACACTGGGAAGGGACCGTTTTTGACTAAC |  |
| SDP112 | GTTAGTCAAAAACGGTCCCTTCCCAGTGTGATGGATATCTGCAG |  |
| SDP113 | ATGGTCTTTGTAGTCCATCATTCTCAGGAGAGAATTGAGTG |  |
| SDP114 | ATGGACTACAAAGACCATGACGGTGATTATAAAGATC  ATGACATCGACTACAAGGATGACGATGACAAGCTTGGAGA  ACATACATCTGGC |  |
| SDP115 | CCGTGCCAACTCATGACC |  |
| SDP116 | GGATGTCGGCAATGAATTCAG | *GAL7-3xFLAG-Cdc20* integration confirmation |
| SDP162 | AGCCTCATCCGACTTCACAG | *GAL7-3xFLAG-SCC1* tagging overlap |
| SDP163 | GCAGATATCCATCACACTGGCCAACACGTACACGTCAACC |  |
| SDP164 | GGTTGACGTGTACGTGTTGGCCAGTGTGATGGATATCTGC |  |
| SDP165 | CACCGTCATGGTCTTTGTAGTCCATTCTCAGGAGAGAATTG  AGTGC |  |
| SDP166 | ATGGACTACAAAGACCATGACGGTGATTATAAAGATCATGA  CATCGACTACAAGGATGACGATGACAAGATCTTGAACGAAC  TGATCAAGTC |  |
| SDP167 | CCATCTCGACATCATAGTCGTC |  |
| SDP168 | CCTTACGACCACAACCAGC | *GAL7-3xFLAG-SCC1* integration confirmation |
| SDP191 | TATGACCTAGAACTGACAGACTCG | *GAL7-3xFLAG-AURK B^IPL1^*  *tagging overlap* |
| SDP192 | GCAGATATCCATCACACTGGGGAGGCCAAATCTGGAGG |  |
| SDP193 | CCTCCAGATTTGGCCTCCCCAGTGTGATGGATATCTGC |  |
| SDP195 | ATGGACTACAAAGACCATGACGGTGATTATAAAGATCATGACATCGACTACAAGGAT GACGATGACAAGTCCTCGCAGAATGTTTCTGG |  |
| SDP196 | CTCGTCAAATCTGCCTAATCTGG |  |
| SDP197 | GTCCTGTAGAAGTTGAATTGC | *GAL7-3xFLAG-AURK B^IPL1^*  integration confirmation |
| SDP170 | TTCGTCGACAACGCTAGCTAATGGCGACGATAATGATG | *SGO1* 3'UTR cloning for complementation |
| SDP171 | TATGGGCCCTGCACTTGCGATAGATATCG |  |
| SDP172 | ACTGTTAACATGTCCCAGAGGCAAGGC | *SGO1* gene amplification for sgo1 deletion complementation |
| SDP173 | GTTGCTAGCTTACTGCAAGACTCCAACGGC |  |
| SDP198 | CCCATCAGGTTTCCGCATGGCTCTTTCGAAATTATCAGCTAACG | *sgo1-K382A* allele amplification for sgo1 deletion complementation |
| SDP199 | CGTTAGCTGATAATTTCGAAAGAGCCATGCGGAAACCTGATGGG |  |
| SDP201 | CTGAAGGAACTGGAAGAGG | *sgo1-K382A* sequencing primer |
| SDP212 | GTGACTAGTAGGCGCTCTTCTGTCGAG |  |
| SDP213 | TCAGGTACCCTACTTGTCATCGTCATCCTTGTAGTCGATGTCATGATCTTTATAATCACCGTCATGGTCTTTGTAGTCAAGCTTCCTTGAAGTCGTAG | *bub1-kd* homology cloning primers |
| SDP214 | ATGTCTTCCGGTGGCAAAGGC | *h2a-T121A* overlap cassette primers |
| SDP215 | GCCTTGGCCTTGCCCTTAGCCTATGATATACATCAGCTGCTGATC |  |
| SDP216 | GATCAGCAGCTGATGTATATCATAGGCTAAGGGCAAGGCCAAGGC |  |
| SDP217 | GATATCCATCACACTGGCGCATCTCCTACTCATCGGCCATCAG |  |
| SDP218 | CTGATGGCCGATGAGTAGGAGATGCGCCAGTGTGATGGATATC |  |
| SDP219 | GGTTCATCAACAATTGTAATTCTCGGTTTATCTGTATTAACAC |  |
| SDP220 | GTGTTAATACAGATAAACCGAGAATTACAATTGTTGATGAACC |  |
| SDP221 | GATCTCCTCTGTGTCGGACATG |  |
| SDP222 | CCATCCAACAAGTCTTTCC | *h2a-T121A* sequencing and confirmation primer |
| SDP223 | CTCCACACAAGAGACTCC | *bub1-kd-3xFLAG* confirmation primers |
| SDP224 | CAGCTATGACCATGATTACG |  |
| KBP001 | ACGGAGCTCGACGTCGTACAAATGCATAC | *GFP-PP1* N-terminal tagging |
| KBP002 | GGCCCATGGTTTATACGAAGTACGTTATATGTG | *GFP-PP1* N-terminal tagging |
| KBP003 | TATACTAGTATGGGAGAACAGCCTGAAATTG |  |
| KBP005 | ATCACTAGTCAATAAACAAGCTCACTTGGAAGG |  |
| KBP006 | GCCGCCGTATCCACCGTATTTC |  |
| VYP51 | TTGAATCCTGCAGATCACAC | *CEN5* qPCR primers |
| VYP52 | CAGCACAGTCAGTTTCATG |  |
| VYP67 | CAGACCCTTCCTTCAGCCG | *CEN2* qPCR primers |
| VYP68 | TGGCAAGGAGTCGTCAGCG |  |
